# Capturing CO_2_ under Dry and Humid Conditions: When Does the Parent MOF Outperform the MTV MOF?

**DOI:** 10.1021/acs.inorgchem.5c02921

**Published:** 2025-09-10

**Authors:** Chunyu Huang, Seyyed Abbas Noorian Najafabadi, Jelco Albertsma, Willy Rook, Marcus Fischer, Martin Hartmann, Monique Ann van der Veen

**Affiliations:** † Chemical Engineering Department, 2860 Delft University of Technology, 2629 HZ Delft, The Netherlands; ‡ Department of Chemical Sciences, 9308 University of Padova, 35131 Padova, Italy; § Erlangen Center for Interface Research and Catalysis (ECRC), 9171 Friedrich-Alexander-Universität Erlangen-Nürnberg (FAU), 91058 Erlangen, Germany

## Abstract

A key challenge in capturing CO_2_ from postcombustion gases is humidity due to competitive adsorption between CO_2_ and H_2_O. Multivariate (MTV) metal–organic frameworks (MOFs) have been considered a promising option to address this problem, e.g., combining CO_2_-affinitive and hydrophobic groups. Here, we synthesized a series of amine and methyl cofunctionalized MTV MIL-53­(Al)-*x*NH_2_(1 *– x*)­CH_3_ and their parent materials. All the mixed linker MIL-53­(Al)-*x*NH_2_(1 – *x*)­CH_3_ showed amino linker enrichment compared to the synthesis ratio, yet the linkers were distributed relatively homogeneously from the bulk to the surface. Material hydrophobicity or hydrophilicity varied with methyl or amino group content, respectively. The single-component adsorption indicated that certain mixed linker MIL-53­(Al)-*x*NH_2_(1 – *x*)­CH_3_ might outcompete the parent materials. In CO_2_–H_2_O competitive adsorption, however, the hydrophobic parental MIL-53­(Al)–CH_3_ outperformed the mixed linker MOFs. CO_2_ adsorption capacities of 5.4, 4.9, and 3.6 wt % were found for 0.3 bar of CO_2_ under 0, 5, and 10% RH, respectively. The results highlight that materials with enhanced hydrophobicity and tight-fitting pores can outperform groups with high CO_2_ affinity in the CO_2_ capture under humid conditions.

## Graphical abstract

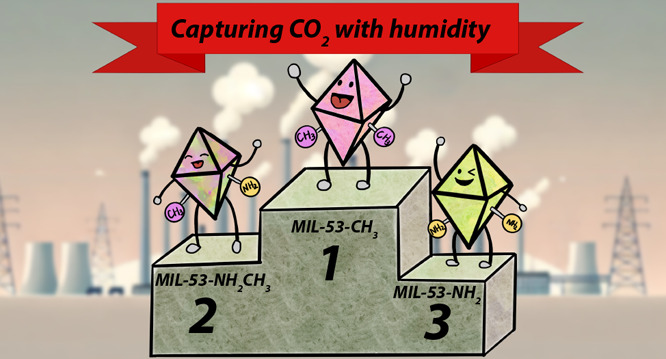

## 1 Introduction

Climate change has become one of the most intractable problems on the planet. One of the main culprits is carbon dioxide (CO_2_), with an emission rate of more than 36 gigatons per year, posing an urgent need for capture and storage. About 45% of the CO_2_ emissions originate from industries and power plants via fuel combustion.^1^ Capturing CO_2_ on solid adsorbents from the exhaust gas stream appears to be a promising alternative technology because it is more cost-effective, has a lower energy demand, and is noncorrosive compared to the conventional way of absorbing CO_2_ by alkanolamine solutions.^2^

A variety of solid adsorbents have been explored for CO_2_ adsorption in the last 20 years, such as zeolites, silicas, carbons, and metal–organic frameworks (MOFs). Among the candidates, MOFs have drawn growing attention because of their high porosities and chemical tailorability.^1^ Depending on the specific industry fields, the postcombustion flue gases can contain 5–20 vol% of moisture.^3^ If precondensation of water content can be avoided, the CO_2_ capture process will be more energy-efficient, making it the key to identifying sorbents that can competitively adsorb CO_2_ in the presence of water.^4,5^ In some cases, cooperative H_2_O–CO_2_ adsorption was even reported, where adsorbed H_2_O enhances CO_2_ adsorption under specific relative humidity.^2^ This type of adsorption leads to moisture-enhanced CO_2_ capture, such as in NOTT-400,^6^ NOTT-401,^7^ MIL-53­(Al),^8^ and TAPB-NDA covalent organic framework (COF).^9^ In these cases, moisture enhancement of CO_2_ only occurs when a limited amount of water is adsorbed and no longer occurs at higher relative humidities where pore-filling water condensation has taken place. However, most frequently, competitive H_2_O–CO_2_ adsorption was observed in MOFs, where H_2_O has a detrimental effect on CO_2_ adsorption due to the large dipole moment and hydrogen bonding potential of H_2_O molecules.^4^ Alkyl amine-based MOFs do not suffer from H_2_O–CO_2_ competitive adsorption but exhibit a high heat of absorption (∼60 to 90 kJ/mol), which is excessive for point-source CO_2_ capture. In contrast, MOF functionalized with aromatic amines presents lower CO_2_ adsorption enthalpies (∼30 to 40 kJ/mol), enabling more energy-efficient regeneration.^10^ However, aromatic amine-based MOFs do suffer from competitive H_2_O adsorption. Zaráte and co-workers^11^ investigated the influence of the amino group in MIL-53­(Al)–NH_2_ on CO_2_ adsorption under humid conditions. The CO_2_ capture ability of MIL-53­(Al)–NH_2_ decreased dramatically in comparison to MIL-53­(Al) with an increase in relative humidity.

To address the challenge of CO_2_–H_2_O competitive adsorption, dedicated efforts have been put into the development of multivariate MOFs (MTV-MOFs), where multifunctionalities, offered by different linkers, metals, or both, can be incorporated into MOF structures. Thus, MTV-MOFs can combine the merits of each parental MOF and have demonstrated synergistic effects.^12^ For example, besides introducing CO_2_-philic groups that suffer from competitive H_2_O adsorption (e.g., aromatic–NH_2_ and phenolic–OH groups), H_2_O repellent groups (e.g., fluoridated, aliphatic groups) can also be introduced simultaneously into the structures. If the relative humidity at which pore-filling water condensation takes place can be increased by employing this strategy while at the same time retaining groups for CO_2_ adsorption, then it could be very favorable. Hu et al.^13^ synthesized a series of mixed linker UiO-66-NH_2_–F_4_, of which UiO-66-NH_2_–F_4_-0.53 can retain 70% of its CO_2_ uptake capacity under 70% relative humidity at 298 K. On the other hand, UiO-66-NH_2_ retained only 12% CO_2_ of its uptake capacity under the same conditions. Similarly, Park et al.^14^ synthesized bifunctionalized MIL-101­(Cr)-NH_2_–F-0.5. It was discovered that MIL-101­(Cr)-NH_2_–F-0.5 only lost 10% capacity for CO_2_ at 303 K and 1 bar under 60% relative humidity, but the capacity was reduced by 40% for MIL-101­(Cr)–NH_2_. In these studies, however, the CO_2_ capturing ability of the hydrophobic parental MOFs under humid conditions was not investigated. Nonetheless, it is necessary to include these results to answer whether developing MTV-MOFs with CO_2_-philic groups and H_2_O repellent groups is truly the best strategy for CO_2_ capture under humid conditions.

Furthermore, only the bulk ratio of different components has generally been investigated in MTV-MOFs and not the spatial distribution, although this also influences the adsorption properties.^15^ A homogeneous structure with various components distributed statistically is usually regarded as the default when a one-pot synthesis is carried out. In reality, however, due to different reactivities of the components affecting the crystallization kinetics, nonrandom structures might form (e.g., cluster domains, and core–shell).^16,17^ Sometimes, even mixed-phase MOFs formed instead of mixed crystallites.^18^ To sum up, the distribution of various components in the MOF crystals is desired for a deeper understanding in the current research.

We focus here on MIL-53 (MIL = Matriaux de l’Institut Lavoisier), an MOF composed of chains of [MO_4_(OH)_2_] polyhedra of inorganic trivalent metal ions (M = Al, Fe, Ga, Cr, and In) and terephthalate linkers,^19^ resulting in one-dimensional diamond-shaped channels. Upon external stimulus, such as temperature, pressure, and/or guest molecule inclusion, the structure can undergo a reversible phase transition between a large pore (LP) form and a narrow pore (NP) form, the so-called ”breathing effect.” The breathing effect can also be influenced by the building blocks and particle size of the MOFs, such as the metal ions and the functionalization of linkers.^19–22^ The combination of the breathing effect of MIL-53 and the mixed-linker strategy enables more possibilities for material design and applications.^23–28^ Yang et al. synthesized a series of mixed linker MIL-53­(Al)–OH_*x*_.^26,27^ The CO_2_ adsorption capacities of MIL-53­(Al)–OH_25_ and MIL-53­(Al)–OH_50_ were approximately 19% higher than that of MIL-53­(Al) under the same conditions. However, MIL-53­(Al)–OH_75_ and MIL-53­(Al)–OH_100_ exhibited much lower CO_2_ uptake due to the introduction of hydroxyl groups on the organic linkers, which stabilized the NP form and made it more difficult for CO_2_ to be absorbed. We report the first MTV-based MIL-53 in which both a functional group targeting CO_2_ adsorption (namely, aromatic −NH_2_) and a functional group enhancing hydrophobicity (namely, −CH_3_) are incorporated. A series of mixed linker MIL-53­(Al)-*x*NH_2_(1 – *x*)­CH_3_ was synthesized, where *x* and (1 – *x*) represent the molar ratio of 2-amino terephthalic acid and 2-methyl terephthalic acid in the initial synthesis (*x* = 0, 0.05, 0.10, 0.25, 0.50, 0.75, and 1), respectively. We explicitly investigated the bulk and the surface concentration of the linker ratio as a measure of spatial distribution across the MOFs through a series of techniques (i.e., elemental analysis, ATR-IR, and XPS). We performed single-component CO_2_ and H_2_O adsorption, as well as evaluated the CO_2_–H_2_O coadsorption for the mixed-linker series and compared these not only to MIL-53­(Al)-NH_2_ but also to the hydrophobic parent MOF MIL-53­(Al)–CH_3_. Intriguingly, we found that the parental MIL-53­(Al)–CH_3_ outperformed the mixed linker MIL-53­(Al)­s to capture CO_2_ under humid conditions.

## 2 Experimental Section

### 2.1 Chemicals and Reagents

Aluminum nitrate nonahydrate (Al­(NO_3_)_3_.9H_2_O) (>98%, Sigma-Aldrich), 2-aminoterephthalic acid (H_2_BDC–NH_2_) (99%, Thermo scientific), 2-methylterephthalic acid (H_2_BDC–CH_3_) (97%, Fluorochem), terephthalic acid (H_2_BDC) (98%, Aldrich), N,N-dimethylformamide (DMF) (>99.9%, Sigma-Aldrich), and acetone (>99.5%, Honeywell) were used without further purification.

### 2.2 Synthesis of MIL-53­(Al)-*x*NH_2_(1 – *x*)­CH_3_

Two pure-linker samples, namely MIL-53-NH_2_ and MIL-53-CH_3_, and five mixed-linker samples MIL-53­(Al)-*x*NH_2_(1 – *x*)­CH_3_(*x* = 0.05, 0.10, 0.25, 0.50, 0.75, and 1) were synthesized based on the literature with some modifications (Scheme 1).^29^

**Scheme 1:**
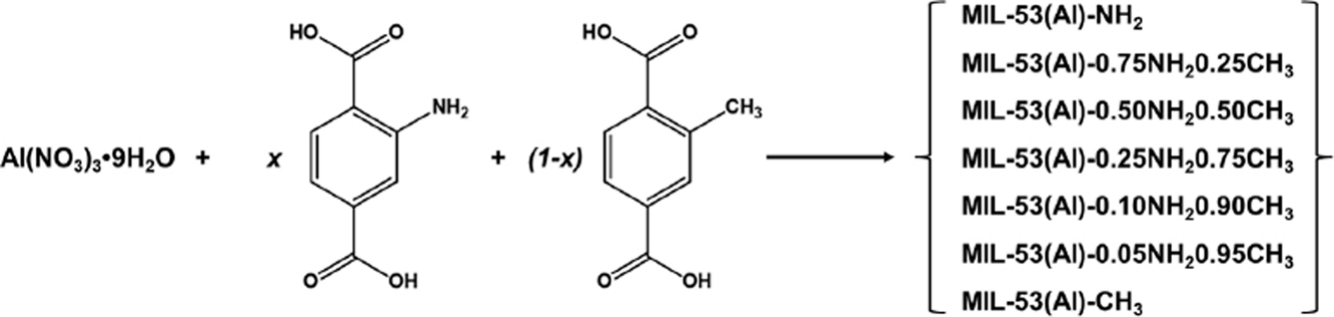
Synthesis of MIL-53­(Al)-*x*NH_2_(1 – *x*)­CH_3_

Briefly, 2 mmol (0.7602 g) of Al­(NO_3_)_3_.9H_2_O was dissolved in 5 mL of distilled water (solution A). Then, 3 mmol of two linkers was added, consisting of various ratios of 2-amino terephthalic acid (H_2_BDC–NH_2_) and 2-methyl terephthalic acid (H_2_BDC–CH_3_). The H_2_BDC–NH_2_ to H_2_BDC–CH_3_ ratio is 0–100% (0 mmol–3 mmol), 5–95% (0.15 mmol–2.85 mmol), 10–90% (0.3 mmol–2.7 mmol), 25–75% (0.75 mmol–2.25 mmol), 50–50% (1.5 mmol–1.5 mmol), 75–25% (2.25 mmol–0.75 mmol), and 100–0% (3 mmol–0 mmol). The linker(s) were added to 20 mL of DMF (solution B). Next, solutions A and B were combined into a 40 mL Teflon-lined autoclave and stirred at 150 rpm for 60 min on a magnetic stirrer plate. Afterward, the autoclaves were placed in an oven at 150 °C for 24 h.

After cooling down to room temperature, the products were washed with DMF three times in a centrifuge, dried in a vacuum oven at 60 °C for 24 h, and marked as as-synthesized samples. Subsequently, about 0.25 g of each product was boiled with 20 mL of DMF in 40 mL Teflon-lined autoclaves at 150 °C for 5 h to remove the unreacted linkers trapped in the pores. Finally, the products were activated by washing with acetone three times in a centrifuge and dried in a vacuum oven at 150 °C for 24 h. These samples are marked as activated samples. The masses of the activated MIL-53­(Al)-*x*NH_2_(1 – *x*)­CH_3_ samples are approximately as follows: 287 mg for MIL-53­(Al)–CH_3_, 250 mg for MIL-53­(Al)-0.05NH_2_0.95CH_3_, 274 mg for MIL-53­(Al)-0.10NH_2_0.90CH_3_, 259 mg for MIL-53­(Al)-0.25NH_2_0.75CH_3_, 270 mg for MIL-53­(Al)-0.50NH_2_0.50CH_3_, 274 mg for MIL-53­(Al)-0.75NH_2_0.25CH_3_, and 260 mg for MIL-53­(Al)-NH_2_. The benchmark MIL-53­(Al) was synthesized and activated using the same protocol mentioned earlier but using 3 mmol terephthalic acid as a linker. The mass of the activated MIL-53­(Al) is about 240 mg.

## 3 Results and Discussion

To confirm that MIL-53­(Al) isostructures have been synthesized, powder X-ray diffraction (PXRD) was first performed on the MIL-53­(Al)-*x*NH_2_(1 – *x*)­CH_3_ series and MIL-53­(Al). As shown in Figures 1 and S1, after activation, all MIL-53­(Al)-*x*NH_2_(1 – *x*)­CH_3_ and MIL-53­(Al) exhibited the characteristic peaks of MIL-53 in a narrow pore form (2θ = 9.4 and 12.3°). Intriguingly, with an increasing amount of −CH_3_ groups incorporated into the structures, two small shoulder peaks at 8.8 and 1 5° can be observed more obviously, which correspond to the large pore form of MIL-53. Crystal parameters of two mixed-linker MOFs (MIL-53­(Al)-*0.75*NH_2_ *0.25*CH_3_ and MIL-53­(Al)-*0.05*NH_2_ *0.95*CH_3_) were then obtained via 3D electron diffraction (Figure S2 and Table S1). The unit cell volume of the amino-rich material is smaller than that of the methyl-rich material due to the hydrogen bonding between −NH_2_ groups and -μ­(OH) in the organic chain, which is in line with the observations in the literature.^20,30^ Thermogravimetric analysis (TGA) showed decent thermal stability of the materials. The materials do not collapse until 400–450 °C, depending on the ratio of −NH_2_ and −CH_3_ groups, which is in agreement with what has been reported in the literature.^30,31^ Before the material decomposition, only a drop around 100 °C was observed due to the evaporation of water molecules, demonstrating that good activation was achieved, meaning no DMF or extra linkers were trapped inside the MOF pores (Figure S3). As shown in Figure 2A, no significant infrared absorption peaks related to free linkers (−COOH at 1680 cm^–1^) or N,N-dimethylfomamide (e.g., C=O stretching at 1665 cm^–1^) appeared, further substantiating proper activation. Scanning electron microscopy (SEM) revealed that the synthesized MIL-53­(Al)-*x*NH_2_(1 – *x*)­CH_3_ and MIL-53­(Al) are highly agglomerated nanoparticles, with a size distribution between 100 and 140 nm (Figure S4), which is comparable to the crystal size reported in the literature when using DMF-water mixed solvents for the synthesis.^31^

**Figure 1:**
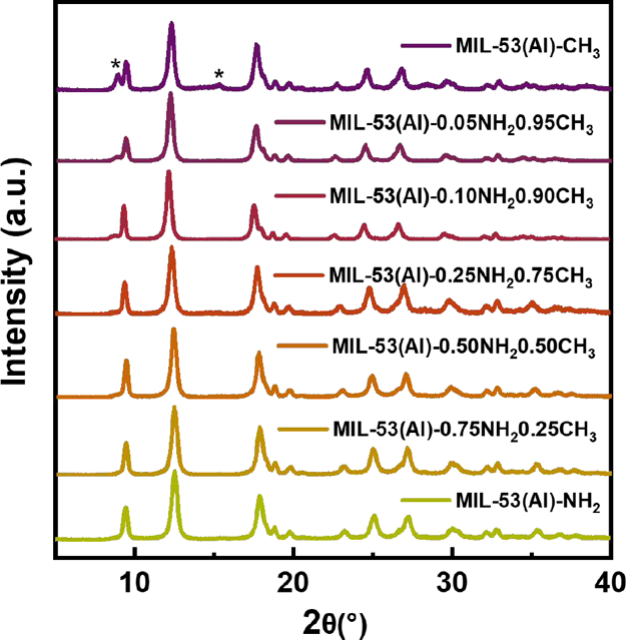
PXRD pattern of activated MIL-53­(Al)-*x*NH_2_(1 – *x*)­CH_3_ series. * corresponds to a diffraction peak of the large pore form.

**Figure 2:**
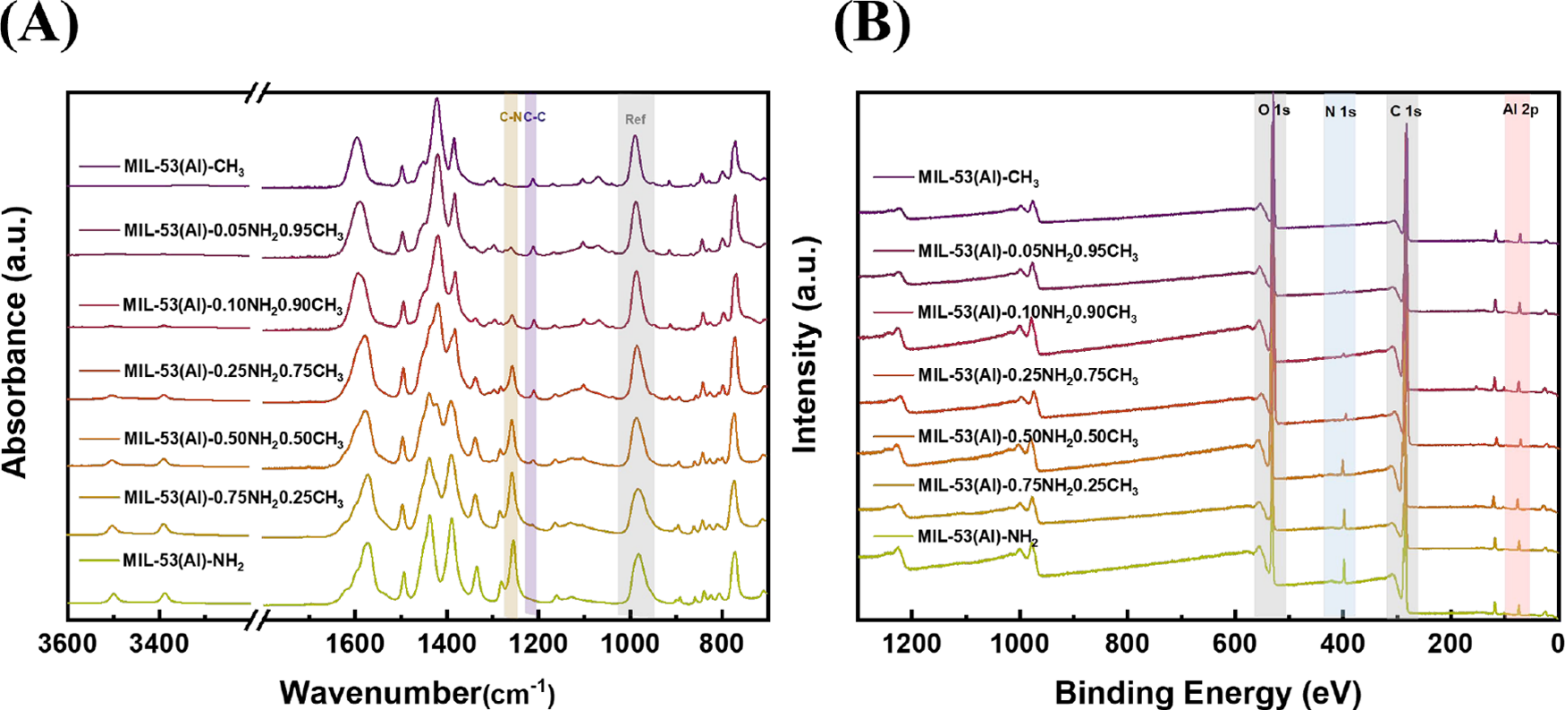
(A) ATR-IR spectra and (B) survey XPS scans on MIL-53­(Al)-*x*NH_2_-(1 – *x*)­CH_3_.

The ratio and distribution of linkers in MTV-MOFs can greatly affect the properties of the materials.^15^ Thus, attenuated total reflectance-infrared spectroscopy (ATR-IR), elemental analysis, and X-ray photoelectron spectroscopy (XPS) were employed to determine the overall ratio of the two linkers in the material as well as the ratio close to the external surface. As shown in Figure 2A, with the ratio varying between two linkers, relative intensity changes in several mid-IR absorption bands could be noticed. With the increasing amount of H_2_BDC–NH_2_ linkers used in the synthesis, bands stemming from N–H symmetric and asymmetric vibrations at 3499 and 3387 cm^–1^ and C–N stretching at 1255 cm^–1^ increased. On the other hand, when more H_2_BDC–CH_3_ linkers were incorporated into the structures, a weak band at 1209 cm^–1^ gradually appeared, which is due to the increasing presence of the methyl group. The penetration depth of the IR beam when using ZnSe is a few micrometers, meaning the IR beam can effectively penetrate the entire MOF crystals, which are only 100–140 nm in size. That allows us to combine the Lambert–Beer law and an internal reference peak (in this case, μ­(OH) band located at 980–986 cm^–1^)^32^ to calculate the average percentage of H_2_BDC–NH_2_ and/or H_2_BDC–CH_3_ in the bulk material from ATR-IR, as shown in Table 1 (for detailed calculation procedures using ATR-IR results, see Table S2). However, we mention that the most representative and separated band attributed to H_2_BDC–CH_3_ at 1209 cm^–1^ is rather weak, resulting in substantial inaccuracy when quantifying H_2_BDC–CH_3_, especially in samples with low CH_3_ content using ATR-IR. To quantify the actual linker ratio in the crystals, we also employed elemental analysis to validate the H_2_BDC–NH_2_ and H_2_BDC–CH_3_ ratio (Tables 1 and S3). The atomic ratios of N:Al were utilized to calculate the linker ratios. Very interestingly, it is found that the amount of the two linkers in the final structure is different from the proportion of the initial ratio in the synthesis for all mixed linker MIL-53s. A greater amount of H_2_BDC–NH_2_ was incorporated into the structures compared to the synthesis ratio, indicating a higher reactivity of H_2_BDC–NH_2_ compared with H_2_BDC–CH_3_. To maintain the consistency of the paper and avoid confusion, we point out that the linker ratios indicated in the MOF names are the ratios in the synthesis rather than the ratios in the crystals. Next, to assess the homogeneity of the mixed linkers within MIL-53s, XPS was further performed to obtain surface information referring to depths of less than 10 nm.^33^ As depicted in Figure 2B, the XPS survey spectra revealed that all of the samples contain C, O, and Al as expected. For the samples in the presence of H_2_BDC–NH_2_ linkers, N was also detected. Subsequently, high-resolution spectra of N 1s, Al 2p, C 1s, and O 1s spectra were deconvoluted, and their atomic percentages were calculated (Figure S5 and Table S4). Thus, the determined H_2_BDC–NH_2_ percentage from XPS closely resembles the values obtained from elemental analysis and ATR-IR, indicating at least from the center to the surface that the two linkers are distributed relatively homogeneously. Nevertheless, on a smaller scale, some degree of nonrandom heterogeneity or pure-linker crystals may still exist.

**Table 1:** Observed–NH_2_ Ratios in Different MOFs via Elemental Analysis and ATR-IR and XPS Techniques

	–NH_2_ ratio via elemental analysis (%)	observed −NH_2_ via ATR-IR (%)	observed −NH_2_ via XPS (%)
MIL-53-NH_2_	100	100	100
MIL-53-0.75NH_2_0.25CH_3_	88	90	89
MIL-53-0.50NH_2_0.50CH_3_	63	75	72
MIL-53-0.25NH_2_0.75CH_3_	49	37	43
MIL-53-0.10NH_2_0.90CH_3_	18	16	14
MIL-53-0.05NH_2_0.95CH_3_	12	12	10
MIL-53-CH_3_	0	0	0

The nitrogen adsorption isotherms on the different mixed-linker MIL-53 samples, the two parental MIL-53­(Al)–CH_3_ and MIL-53­(Al)-NH_2_, as well as MIL-53­(Al) are shown in Figure 3A. The benchmark MIL-53­(Al) displayed a type I isotherm. However, MIL-53-CH_3_, MIL-53-NH_2_, and mixed linker MIL-53­(Al)-*x*NH_2_-(1 – *x*)­CH_3_ showed an “S”-shaped adsorption isotherms, which is caused by an NP to LP transition.^25,30,34^ The inflection point of the mixed linker MIL-53s is shifted compared to those of the parental MIL-53­(Al)–CH_3_ and MIL-53­(Al)-NH_2_ (Table S5). Moreover, the mixed linker MIL-53 did not behave as a linear combination of the parent MOFs, which indicates the incorporation of both linkers into the same crystals, as shown in Figure S6.

**Figure 3:**
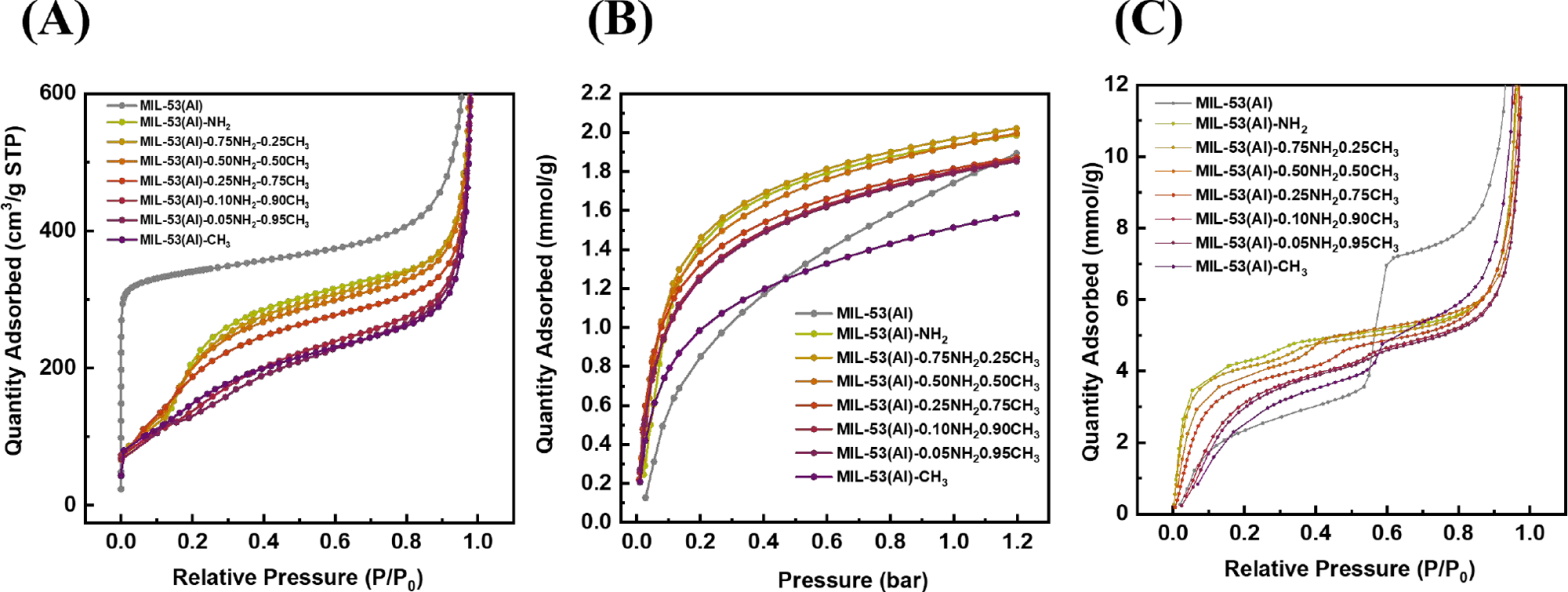
(A) N_2_ adsorption at 77K, (B) CO_2_ adsorption at 298 K, and (C) H_2_O adsorption at 293 K on MIL-53­(Al) and MIL-53­(Al)-*x*NH_2_(1 – *x*)­CH_3_.

CO_2_ adsorption was then carried out to assess the CO_2_ capacity of each MOF (Figure 3B), and individual CO_2_ adsorption–desorption curves are plotted in Figure S9). No phase transition was observed in either MIL-53­(Al) or MIL-53­(Al)-*x*NH_2_(1 – *x*)­CH_3_ during CO_2_ adsorption at 1.2 bar and 298 K. For MIL-53­(Al), a potential low-pressure breathing behavior (LP–NP–LP) can occur below 1 bar, but this transition is highly dependent on crystal size.^35^ In our case, the crystals were too small to exhibit this transformation.^21,22^ For MIL-53­(Al)-*x*NH_2_(1 – *x*)­CH_3_, a pressure-induced transition from the NP to LP form may occur at higher CO_2_ pressures, but the applied pressure (1.2 bar) remained below the threshold required to trigger such a change.^36,37^ Overall, the introduction of the −NH_2_ group presented a positive impact in increasing the total CO_2_ capacity. Specifically, at 1.2 bar, MIL-53­(Al)-0.75NH_2_0.25CH_3_ exhibited a CO_2_ capacity of 2.02 mmol/g (∼0.45 CO_2_ per Al­(OH)­(X-terepthalate)), which is slightly higher than that of MIL-53­(Al)-NH_2_ (1.98 mmol/g, ∼0.44 CO_2_ per Al­(OH)­(X-terepthalate), and higher than that of MIL-53­(Al)–CH_3_ (1.58 mmol/g, ∼0.35 CO_2_ per Al­(OH)­(X-terepthalate)), and MIL-53­(Al) (1.89 mmol/g, ∼0.39 CO_2_ per Al­(OH)­(X-terepthalate)). Notice that 0.5 CO_2_ per Al­(OH)­(X-terepthalate) means that one CO_2_ molecule sits in one diamond-shaped window of four linkers in the NP form.^36^ This is in line with earlier findings that much higher CO_2_ partial pressures are required to transform MIL-53 or MIL-53–NH_2_ into the LP form and thereby utilize their full adsorption capacity.^38^ Intriguingly, up to 0.05 bar, MIL-53­(Al)–CH_3_ had a higher CO_2_ uptake than MIL-53­(Al)-NH_2_; Up to 0.08 bar, all mixed-linker MIL-53­(Al)-*x*NH_2_(1 – *x*)­CH_3_ displayed a higher absorption than MIL-53­(Al)-NH_2_; Up to 0.13 bar, MIL-53­(Al)-0.50NH_2_0.50CH_3_ and MIL-53­(Al)-0.75NH_2_0.25CH_3_ exhibited greater adsorption than MIL-53­(Al)-NH_2_, as shown in Figure S7. This phenomenon may be attributed to the introduction of −CH_3_ groups slightly expanding the unit cell, yet leading to a smaller free pore diameter, making a more snug fit for CO_2_, and thus stronger CO_2_ adsorption. Yet, with pressure increasing, the CO_2_ adsorbed among MIL-53­(Al)-0.25NH_2_0.75CH_3_, MIL-53­(Al)-0.10NH_2_0.90CH_3_, and MIL-53­(Al)-0.05NH_2_0.95CH_3_ became similar, attaining 1.2 mmol/g at 1.2 bar. Similarly, the amount of CO_2_ adsorbed by two amino-rich mixed-linker MOFs (MIL-53­(Al)-0.75NH_2_0.25CH_3_ and MIL-53­(Al)-0.50NH_2_0.50CH_3_), and MIL-53­(Al)-NH_2_, all reached approximately 2 mmol/g at 1.2 bar. This leads to the finding that there is a nonlinear relationship between the −NH_2_ amount in crystals and the CO_2_ capacity, meaning the isotherms of mixed linker MIL-53­(Al)-*x*NH_2_(1 – *x*)­CH_3_ are not a weighted superposition of individual isotherms of the parental MOFs based on the linker proportions in the crystals, same as in N_2_ adsorption (see calculated CO_2_ adsorption isotherms in Figure S8).

Another striking observation is that MIL-53­(Al)–CH_3_ also showed slightly higher CO_2_ uptake in comparison with MIL-53­(Al) below 0.4 bar. We hypothesize that additional methyl groups inside the pores might decrease the free pore diameter, which increased van der Waals interactions without compromising the Coulombic interactions.^39,40^ All in all, this suggests that a higher concentration of −NH_2_ groups within the pores does not necessarily result in superior materials in terms of CO_2_ capturing ability.

Next, to understand the hydrophobicity/hydrophilicity change with the variance of H_2_BDC–NH_2_ and H_2_BDC–CH_3_ linkers, we performed water adsorption measurements on the materials (Figure 3C), and for individual water adsorption isotherms, see Figure S10). The large additional water uptake for *P*/*P*_0_ > 0.8 is due to water condensation in the voids between the MOF particles and is excluded from further discussion here. The benchmark MIL-53­(Al) displays a type IV isotherm. Water is absorbed up to 3.4 mmol/g at 0.5 *P*/*P*_0_ (∼0.70 H_2_O per Al­(OH)­(X-terephtalate)), followed by a sudden uptake to 7.1 mmol/g (∼1.49 H_2_O per Al­(OH)­(X-terephtalate)) around 0.6 *P*/*P*_0_. This step corresponds to a phase transition from NP to LP form, which is in line with what has been reported previously when the MIL-53­(Al) was synthesized in DMF.^41–43^ In contrast, all MIL-53­(Al)-*x*NH_2_(1 – *x*)­CH_3_ showed a substantially lower uptake compared to that of MIL-53­(Al) at 0.8 *P*/*P*_0_, indicating that the introduction of functional groups stabilized the structures in the NP form during the water adsorption process. Water molecules filled up the pores in the low-pressure region up to 4.5 mmol/g for MIL-53­(Al)-NH_2_, followed by a small second step between 0.25 and 0.55 *P*/*P*_0_ with an uptake of 4.87 mmol/g (1.09 H_2_O per Al­(OH)­(X-terephtalate)), which is comparable to the reported water adsorption capacity of MIL-53­(Al)–NH_2_ by Yamada et al.^43^ As expected due to the nature of the functional groups, MIL-53­(Al)–CH_3_ and MIL-53­(Al)–NH_2_ are the most hydrophobic and hydrophilic materials, respectively, in terms of the initial slope of the water adsorption isotherm and total water uptake. The mixed linker MIL-53­(Al)-*x*NH_2_(1 – *x*)­CH_3_ shows transient isotherms between the two parental materials; the more the −CH_3_ groups are incorporated into the structures, the more hydrophobic the materials become, and *vice versa*.

As the CO_2_ adsorption capacity is only impacted by a decrease in −NH_2_ content to a limit (even with up to 95% −CH_3_ in the synthesis), while the material does get more hydrophobic with increasing −CH_3_ content, we expect that certain ratios of −CH_3_/–NH_2_ mixed linker MIL-53 will have a higher CO_2_ adsorption under humid conditions compared to the parent materials, especially those with high −CH_3_ content. Therefore, competitive CO_2_–H_2_O adsorption measurements were performed on the two pure-linker MOFs (MIL-53­(Al)–NH_2_ and MIL-53­(Al)–CH_3_), and two mixed-linker MOFs (MIL-53­(Al)-0.05NH_2_0.95CH_3_ and MIL-53­(Al)-0.25NH_2_0.75CH_3_). After being dried in situ, the materials were first equilibrated at certain relative humidities, and then experienced a CO_2_ adsorption–desorption cycle at 298 K (for kinetic data, please see Figures S11–S14). The weight change between the prehumidified sample and after CO_2_ loading was then considered as CO_2_ being adsorbed, assuming that CO_2_ did not desorb the preloaded water. Then, the uptake of CO_2_ with exposure to different humidity can be calculated using eq 1, where *M*_CO_2_+H_2_O_ represents the equilibrated weight after uptaking CO_2_ and H_2_O, *M*_H_2_O_ represents the equilibrated weight after uptaking H_2_O, and *M*_0_ is the weight of the dried material.

$${\mathrm{CO}}_{2}\;\mathrm{uptake}\;(\mathrm{mass}\;\%)=\frac{M_{{\mathrm{CO}}_{2}+H_{2}O}-M_{H_{2}O}}{M_{0}}$$ 1

As shown in Figure 4, it can be noticed that under dry conditions, MIL-53­(Al)-NH_2_ has the highest CO_2_ uptake when CO_2_ concentration is higher than 5 vol %, whereas the other three materials had better performance at 5 vol %, which is also well in line with the results from volumetrically measured CO_2_ adsorption isotherms, displayed in Figures 3A and S7. At 5% relative humidity and 30 vol % CO_2_, MIL-53­(Al)-NH_2_ already lost 82% of its original adsorption capacity. In fact, for all measured relative humidity (RH), the higher the −CH_3_ content, the higher the CO_2_ adsorption, meaning that MIL-53­(Al)–CH_3_ outperformed all mixed-linker materials for all humidities. At 5 and 10% RH, in the presence of 30 vol % CO_2_, MIL-53­(Al)–CH_3_ retains ∼91 and ∼65% of its original CO_2_ adsorption capacity, respectively. For higher relative humidities, the CO_2_ adsorption capacity quickly deteriorates for all materials. The mixed-linker MOFs show transient behavior between the two parent materials: under 5% RH and 30 vol % CO_2_, 55 and 72% capacity were still remained for MIL-53­(Al)-0.25NH_2_0.75CH_3_ and MIL-53­(Al)-0.05NH_2_0.95CH_3_, respectively. In fact, for MIL-53­(Al)–CH_3_, quite significant CO_2_ adsorption capacities of 4.9 and 3.6 wt % were found for 30 vol % CO_2_ and 5 and 10% RH, respectively. This shows that −NH_2_ groups in the structures had a negative impact on capturing CO_2_ under humid conditions, implying that H_2_O molecules had a stronger affinity than CO_2_ to competitively bond with −NH_2_ sites, which is consistent with the findings of Zárate et al.^11^ Sánchez−Serratos et al. have reported the CO_2_–H_2_O competitive adsorption on MIL-53­(Al), where a cooperative CO_2_–H_2_O adsorption was observed.^8^ They discovered a 1.5-fold CO_2_ adsorption increase, up to 5.2 wt % under 20% RH at 30 °C compared with dry CO_2_.

**Figure 4:**
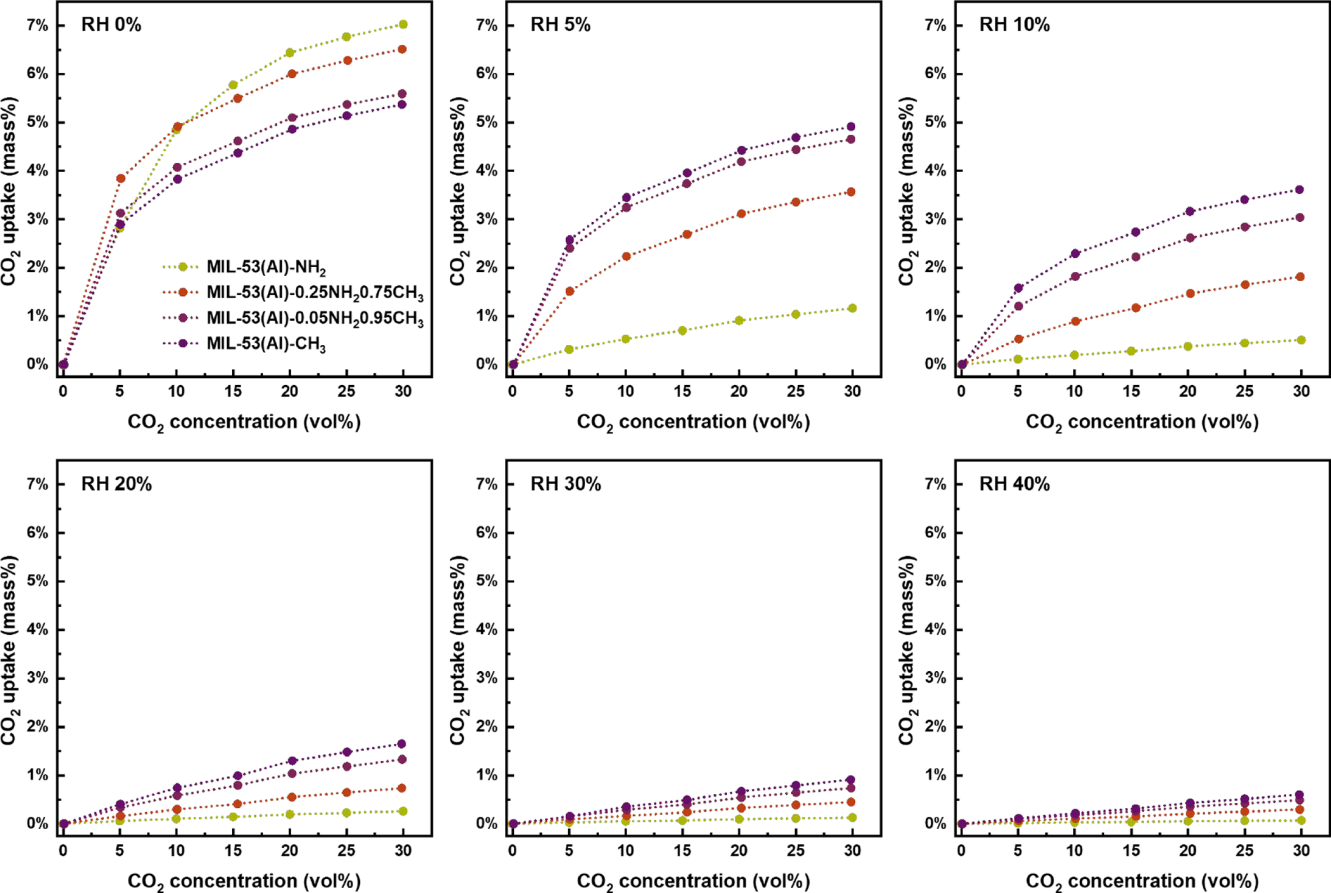
Mass change attributed to CO_2_ uptake under 0, 5, 10, 20, 30, and 40% RH at 298 K on MIL-53­(Al)–NH_2_, MIL-53­(Al)-0.25NH_2_0.75CH_3_, MIL-53­(Al)-0.05NH_2_0.95CH_3_, and MIL-53­(Al)–CH_3_. The dashed lines were added for eye guidance.

We also expect that introducing hydrophobic groups, e.g., fluorine groups, into MIL-53­(Al) may help mitigate water interference during CO_2_ capture. Based on the water adsorption results of MIL-53­(Al)-F_2_ reported by Van Der Voort and co-workers^44^ and that of MIL-53­(Al)-F_4_ reported by Guiotto et al.,^45^ the steep water uptake step corresponding to water cluster formation is shifted to 50–70% RH upon fluorination, compared to ∼10% RH for MIL-53­(Al). Therefore, it can be expected that the CO_2_ adsorption capacity of MIL-53­(Al)-Fx would be affected by water vapor only to a very limited extent prior to the pore-filling step. The strong CO_2_ adsorption in MIL-53­(Al)–CH_3_ is rationalized by the small pore size in the NP form of this MOF. A similar strong CO_2_ adsorption in MIL-53­(Al)-Fx will thus depend on the structure remaining in the NP form under the relevant conditions. A caveat, however, is that compounds containing fluorinated carbon are under increased scrutiny due to their bioaccumulation and persistence in the environment due to the high stability of the fluorine-carbon bond. In summary, the competitive CO_2_–H_2_O adsorption behavior highlighted the importance of enhancing the hydrophobicity of adsorbents for carbon capture in the presence of water. It underscores for MTV-MOFs that a proper comparison with the parent MOFs is warranted.

## 4 Conclusions

In this study, a series of mixed linker MIL-53­(Al)-*x*NH_2_(1 *– x*)­CH_3_ and two parental materials were prepared successfully. With a combination of techniques, not only the bulk ratio but also the average spatial arrangement of BDC–NH_2_ and BDC–CH_3_ were studied. It is found that the actual ratios of BDC–NH_2_ in mixed-linker MOFs were much higher than the initial ratio in the synthesis, indicating a higher reactivity of BDC–NH_2_ compared to BDC–CH_3_. By comparing the bulk ratio and surface ratio of the two linkers, we also confirmed a relatively homogeneous linker distribution from core to surface.

For all competitive and single-component CO_2_ and H_2_O adsorption measurements (up to 1.2 bar of CO_2_ and 95% RH), the mixed linker MIL-53­(Al)-*x*NH_2_(1 – *x*)­CH_3_ and two parental materials remained in the NP form. For single-component CO_2_ adsorption, amino groups overall showed a positive impact on increasing the CO_2_ adsorption capacity. Among the materials, MIL-53­(Al)-0.75NH_2_0.25CH_3_ exhibited the highest CO_2_ uptake of 2.02 mmol/g at 1.2 bar at 298 K. However, the increase in CO_2_ uptake is more than linear to the actual number of amino groups in the crystals. The single-component water adsorption isotherms at 293 K indicated that the introduction of methyl groups could effectively postpone the H_2_O uptake to a higher RH, whereas the presence of amino groups made the materials more hydrophilic.

Based on the single-component adsorption behavior, the two mixed-linker MOFs most rich in CH_3_ groups (MIL-53­(Al)-0.05NH_2_0.95CH_3_ and MIL-53­(Al)-0.25NH_2_0.75CH_3_) were judged to be most promising to examine the CO_2_ uptake under humid conditions (RH = 5, 10, 20, 30, and 40%), and compared to the two parent MOFs. However, in the competitive adsorption of CO_2_–H_2_O, no synergetic effect between amino groups and methyl groups was observed. Under the tested RH range, the CO_2_ adsorption for all MOFs decreased compared to dry conditions. Surprisingly, the hydrophobic parental MIL-53­(Al)–CH_3_ outperformed the mixed linker MIL-53­(Al)-*x*NH_2_(1 – *x*)­CH_3_ in terms of the capture of CO_2_ under moist conditions. Notably, considerable CO_2_ adsorption capacities of 4.9 and 3.6 wt % were observed under conditions of 30 vol % CO_2_ with 5 and 10% RH, respectively.

Although tremendous research efforts have been dedicated to developing MTV-MOFs for the purpose of capturing CO_2_ under humid conditions, so far, none of them have compared the MTV-MOFs with the hydrophobic parental one. The results here, however, demonstrate that the hydrophobic parental MOF can have the best performance to capture CO_2_ under humid conditions and should thus be included in future studies. The work highlights that materials with enhanced hydrophobicity and tight-fitting pores rather than hydrophilic groups can be good. Sometimes, for CO_2_ capture under humid conditions the hydrophobic material even outperforms materials with specific sites with high CO_2_ affinity, due to their hydrophilicity. We hope that this study will help steer the direction of material development in this regard.

## Supplementary Material



## Notes

Experimental details, materials, and characterizations of the materials, including TGA, SEM, IR, XPS, N_2_, H_2_O, and CO_2_ adsorption isotherms and competitive CO_2_–H_2_O adsorption data (PDF)
