# Supplementary material for: Capturing CO2 under Dry and Humid Conditions: When Does the Parent MOF Outperform the MTV MOF?
Source: Inorg Chem. 2025 Sep 10;64(37):18916–24. doi: 10.1021/acs.inorgchem.5c02921 (PMC12458701; doi:10.1021/acs.inorgchem.5c02921)
Supplement: Supplementary file 1 [file ic5c02921_si_001.pdf]

Supporting information

Capturing CO_2 under dry and humid conditions: When does the parent MOF outperform the MTV MOF?

Chunyu Huang,[†] Seyyed Abbas Noorian Najafabadi,[†,‡] Jelco Albertsma,[†] Willy Rook,[†] Marcus Fischer,[¶] Martin Hartmann,[¶] and Monique Ann van der Veen[*,†]

†*Chemical Engineering Department, Delft University of Technology, Delft, The Netherlands*

‡*Department of Chemical Sciences, University of Padova, 35131, Padova, Italy*

¶*Erlangen Center for Interface Research and Catalysis (ECRC), Friedrich-Alexander-Universität Erlangen-Nürnberg (FAU), Erlangen, Germany*

E-mail: m.a.vanderveen@tudelft.nl

Phone: +31 15 2786458

Experimental section

Apparatus and Measurements

Powder X-ray diffraction (PXRD) was performed with a Bruker D8 Advanced diffractometer with a Cu Kα source ($\lambda = 1.5418$ Å). The measured 2θ range was $3 - 70°$.

Thermogravimetric analysis (TGA) was carried out using a Mettler Toledo 1600 TGA/SDTA851. The measurements were performed under air with a gas flow of 100 mL/min. A pre-treatment at 30 °C for 30 minutes was employed and then the temperature increased by 10 °C/min up to 800 °C.

Scanning Electron Microscopy (SEM) was performed using a Jeol JSM-IT700HR. The parameters used were indicated in the pictures.

Attenuated Total Reflectance Infrared spectroscopy (ATR-IR) was performed using Nicolet ISQ-50 FT-IR spectrometer, with 256 scans per sample detected by an MCT/A detector using a ZnSe crystal with a step size of 1cm^{-1} over a range of 4000 - 400 cm^{-1}.

X-ray photoemission spectroscopy (XPS) measurements were performed using Thermofisher K-Alpha using an Al Kα source with the cathode set to 12 kV and the beam current to 3 mA. A flood gun was used to minimise the effects of sample charging. The pass energy was set to 140 eV for the survey scans and 50 eV for the elemental scans. Fitting of the elemental scans was done in the Avantage software. A smart background was used to obtain the baseline which was used to fit background corrected peaks to obtain the surface elemental composition.

For 3D-ED measruements, samples were measured on the XtaLAB Synergy-ED. The sample was dispersed in ethanol, sonicated, and drop-casted on lacey-carbon supported copper TEM grids. The datasets were measured from different crystallites with a wavelength of 0.0251 Å at 175 K. In combination with cryo-transfer (*viz.* freezing samples prior to introduction to vacuum), solvate and hydrate structures remain unscathed.

Elemental analysis was used to determine the linker ratio in the mixed linker MIL-53s. The

analysis was performed by Mikroanalytisches Laboratorium Kolbe. An Elementar Model Vario Mikro CHNS analyser (for C, H, and N), and for the metals a Spectro Model Spectro Acros ICP after microwave digestion on a CEM MARS 6.

N_2 adsorption isotherms were obtained using Micrometrics Tristar II at 77 K. For MIL-53-$x$$NH_2$-*(1-x)*$CH_3$, setting were customized as first dosing fixed 35 cm^3/g STP N_2 in order to obtain complete adsorption isotherms. Prior to the N_2 measurements, all samples were degassed under N_2 gas flow at 90 °C for one hour and then at 150 °C for 16 hours.

CO_2 adsorption isotherms were obtained using Micrometrics Tristar II at 298 K from 0 to 120 kPa. Prior to the CO_2 measurements, all samples were degassed under N_2 gas flow at 150 °C for 3 hours.

Water adsorption isotherms were obtained using Micrometrics: 3Flex Surface and Catalyst Characterization at 293 K. Prior to the H_2O measurements, all samples were degassed under vacuum at 150 °C for 16 hours.

CO_2-H_2O competitive adsorption was performed using Dynamic Vapor Sorption (DVS) Carbon Advanced from Surface Measurement Systems. Prior to the competitive adsorption, samples were dried *in situ* at 150 °C for 3 hours. Relative humidities were set at 0%, 5%, 10%, 20%, 30%, and 40%. Detailed parameters and experiment sequences are showing below.

Stage #	Stage Type	dm/dt [%/min]	Partial pressure (A) [%] –H_2O	Partial pressure (B) [%]-CO_2	Incubator Temp [°C]	Carrier Gas
1	dm/dt	0.002000	0	0.00	25	N_2
2	dm/dt	0.002000	0	5.00	25	N_2
3	dm/dt	0.002000	0	10.00	25	N_2
4	dm/dt	0.002000	0	15.00	25	N_2
5	dm/dt	0.002000	0	20.00	25	N_2
6	dm/dt	0.002000	0	25.00	25	N_2
7	dm/dt	0.002000	0	30.00	25	N_2
8	dm/dt	0.002000	0	25.00	25	N_2
9	dm/dt	0.002000	0	20.00	25	N_2
10	dm/dt	0.002000	0	15.00	25	N_2
11	dm/dt	0.002000	0	10.00	25	N_2
12	dm/dt	0.002000	0	5.00	25	N_2
13	dm/dt	0.002000	0	0.00	25	N_2

Stage #	Stage Type	dm/dt [%/min]	Partial pressure (A) [%] –H_2O	Partial pressure (B) [%]-CO_2	Incubator Temp [°C]	Carrier Gas
1	dm/dt	0.002000	5	0.00	25	N_2
2	dm/dt	0.002000	5	5.00	25	N_2
3	dm/dt	0.002000	5	10.00	25	N_2
4	dm/dt	0.002000	5	15.00	25	N_2
5	dm/dt	0.002000	5	20.00	25	N_2
6	dm/dt	0.002000	5	25.00	25	N_2
7	dm/dt	0.002000	5	30.00	25	N_2
8	dm/dt	0.002000	5	25.00	25	N_2
9	dm/dt	0.002000	5	20.00	25	N_2
10	dm/dt	0.002000	5	15.00	25	N_2
11	dm/dt	0.002000	5	10.00	25	N_2
12	dm/dt	0.002000	5	5.00	25	N_2
13	dm/dt	0.002000	5	0.00	25	N_2

Stage #	Stage Type	dm/dt [%/min]	Partial pressure (A) [%] –H_2O	Partial pressure (B) [%]-CO_2	Incubator Temp [°C]	Carrier Gas
1	dm/dt	0.002000	10	0.00	25	N_2
2	dm/dt	0.002000	10	5.00	25	N_2
3	dm/dt	0.002000	10	10.00	25	N_2
4	dm/dt	0.002000	10	15.00	25	N_2
5	dm/dt	0.002000	10	20.00	25	N_2
6	dm/dt	0.002000	10	25.00	25	N_2
7	dm/dt	0.002000	10	30.00	25	N_2
8	dm/dt	0.002000	10	25.00	25	N_2
9	dm/dt	0.002000	10	20.00	25	N_2
10	dm/dt	0.002000	10	15.00	25	N_2
11	dm/dt	0.002000	10	10.00	25	N_2
12	dm/dt	0.002000	10	5.00	25	N_2
13	dm/dt	0.002000	10	0.00	25	N_2

Stage #	Stage Type	dm/dt [%/min]	Partial pressure (A) [%] –H_2O	Partial pressure (B) [%]-CO_2	Incubator Temp [°C]	Carrier Gas
1	dm/dt	0.002000	20	0.00	25	N_2
2	dm/dt	0.002000	20	5.00	25	N_2
3	dm/dt	0.002000	20	10.00	25	N_2
4	dm/dt	0.002000	20	15.00	25	N_2
5	dm/dt	0.002000	20	20.00	25	N_2
6	dm/dt	0.002000	20	25.00	25	N_2
7	dm/dt	0.002000	20	30.00	25	N_2
8	dm/dt	0.002000	20	25.00	25	N_2
9	dm/dt	0.002000	20	20.00	25	N_2
10	dm/dt	0.002000	20	15.00	25	N_2
11	dm/dt	0.002000	20	10.00	25	N_2
12	dm/dt	0.002000	20	5.00	25	N_2
13	dm/dt	0.002000	20	0.00	25	N_2

Stage #	Stage Type	dm/dt [%/min]	Partial pressure (A) [%] –H_2O	Partial pressure (B) [%]-CO_2	Incubator Temp [°C]	Carrier Gas
1	dm/dt	0.002000	30	0.00	25	N_2
2	dm/dt	0.002000	30	5.00	25	N_2
3	dm/dt	0.002000	30	10.00	25	N_2
4	dm/dt	0.002000	30	15.00	25	N_2
5	dm/dt	0.002000	30	20.00	25	N_2
6	dm/dt	0.002000	30	25.00	25	N_2
7	dm/dt	0.002000	30	30.00	25	N_2
8	dm/dt	0.002000	30	25.00	25	N_2
9	dm/dt	0.002000	30	20.00	25	N_2
10	dm/dt	0.002000	30	15.00	25	N_2
11	dm/dt	0.002000	30	10.00	25	N_2
12	dm/dt	0.002000	30	5.00	25	N_2
13	dm/dt	0.002000	30	0.00	25	N_2

Stage #	Stage Type	dm/dt [%/min]	Partial pressure (A) [%] –H_2O	Partial pressure (B) [%]-CO_2	Incubator Temp [°C]	Carrier Gas
1	dm/dt	0.002000	40	0.00	25	N_2
2	dm/dt	0.002000	40	5.00	25	N_2
3	dm/dt	0.002000	40	10.00	25	N_2
4	dm/dt	0.002000	40	15.00	25	N_2
5	dm/dt	0.002000	40	20.00	25	N_2
6	dm/dt	0.002000	40	25.00	25	N_2
7	dm/dt	0.002000	40	30.00	25	N_2
8	dm/dt	0.002000	40	25.00	25	N_2
9	dm/dt	0.002000	40	20.00	25	N_2
10	dm/dt	0.002000	40	15.00	25	N_2
11	dm/dt	0.002000	40	10.00	25	N_2
12	dm/dt	0.002000	40	5.00	25	N_2
13	dm/dt	0.002000	40	0.00	25	N_2

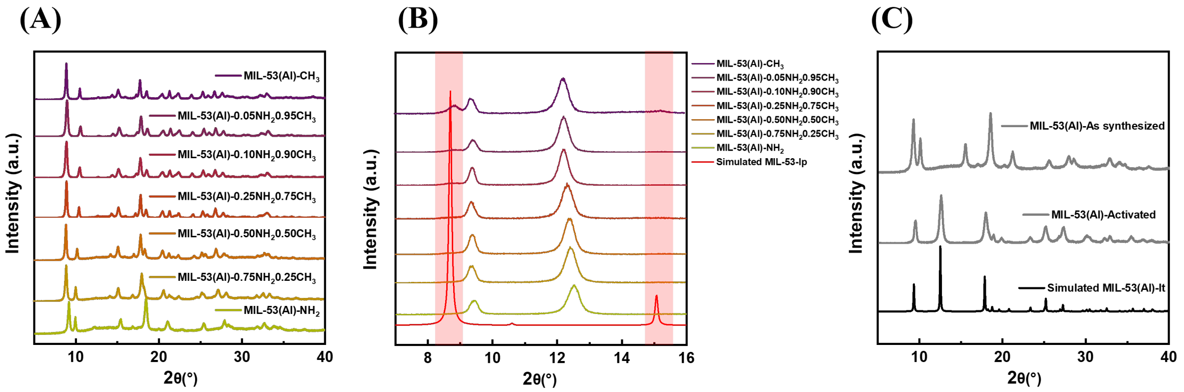

Figure S1. (A) PRXD of as-synthesized MIL-53(Al)-xNH$_2$*(1-x)*CH$_3$. (B) Zoom in PXRD of activated MIL-53(Al)-xNH$_2$*(1-x)*CH$_3$ with simulated MIL-53(Al) large pore form.(C) PXRD of benchmark MIL-53(Al).

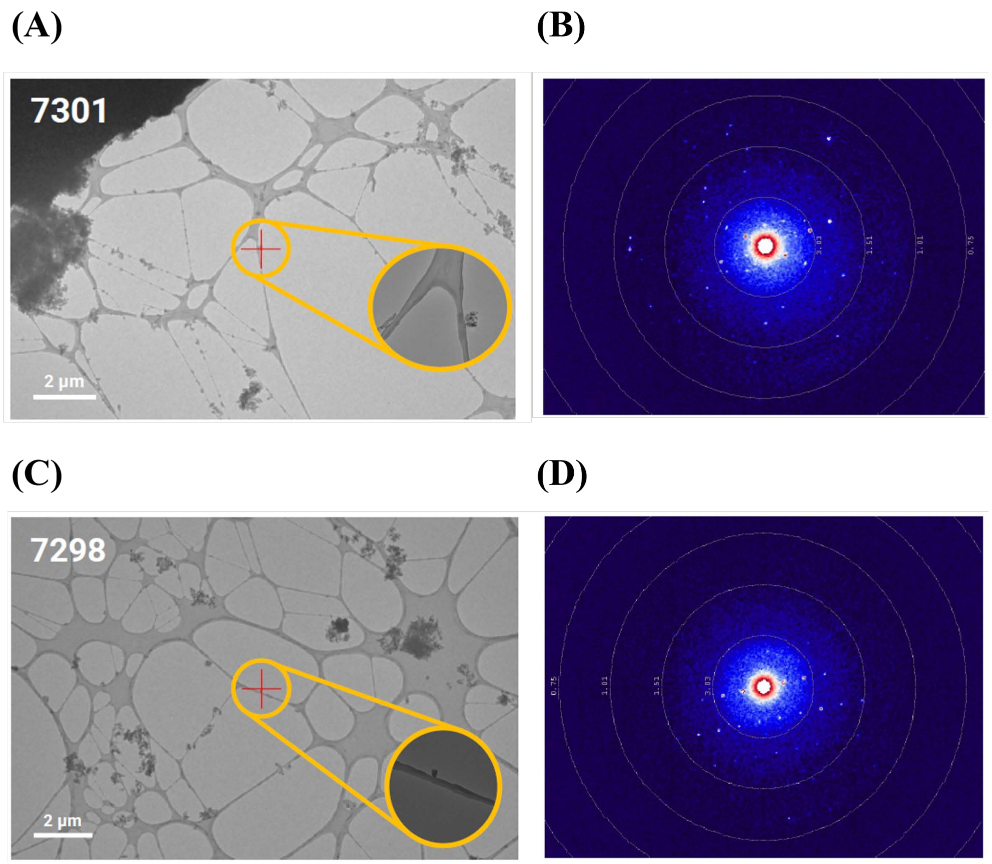

Figure S2. (A) Grain snapshots and (B) exemplary diffraction images of MIL-53(Al)-$\mathit{0.75}$$NH_2$$\mathit{0.25}$$CH_3$; (C) Grain snapshots and (D) exemplary diffraction images of MIL-53(Al)-$\mathit{0.05}$$NH_2$$\mathit{0.95}$$CH_3$.

Table S1. Refined lattice Parameters of MIL-53(Al)-$\mathit{0.75}$$NH_2$$\mathit{0.25}$$CH_3$ and MIL-53(Al)-$\mathit{0.05}$$NH_2$$\mathit{0.95}$$CH_3$ from 3D-ED.

	a (Å)	b (Å)	c (Å)	V ($\mathring{A}^3$)
**MIL-53(Al)-*0.75*NH$_2$*0.25*CH$_3$**	6.533(2)	7.480(3)	18.995(6)	929.1(6)
**MIL-53(Al)-*0.05*NH$_2$*0.95*CH$_3$**	6.5696(12)	7.955(3)	18.855(7)	983.6(5)

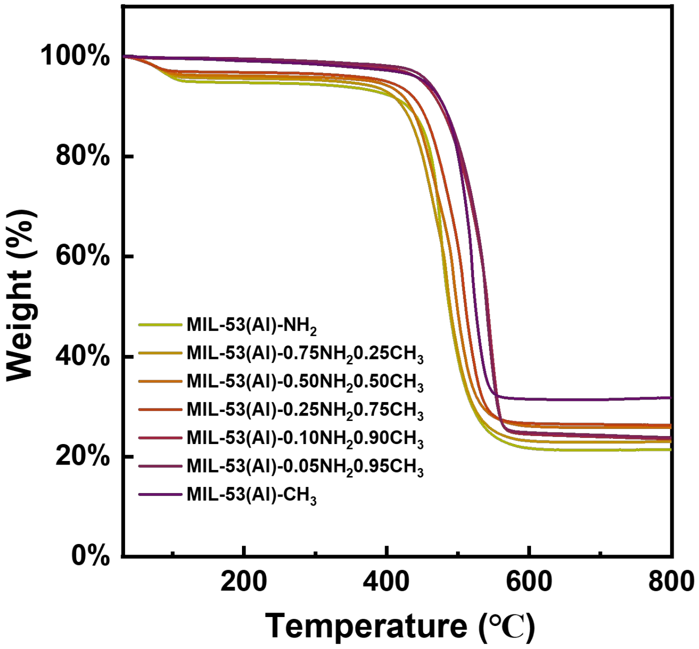

Figure S3. TG analyses under air atmosphere of MIL-53(Al)-$x$$NH_2$*(1-x)*$CH_3$.

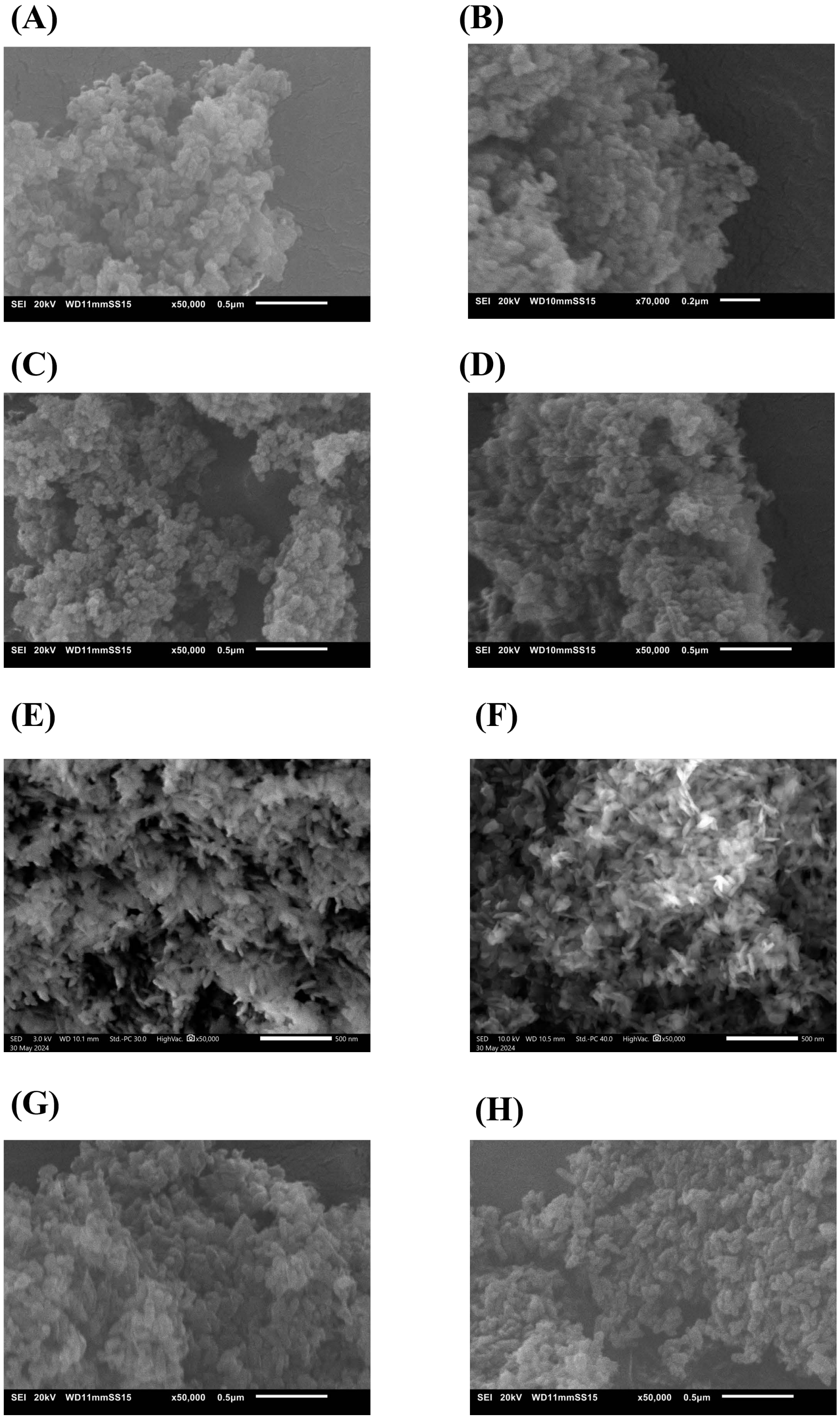

Figure S4. SEM images of (A) MIL-53(Al)-NH_2, (B) MIL-53(Al)-*0.75*NH_2*0.25*CH_3, (C) MIL-53(Al)-*0.50*NH_2*0.50*CH_3, (D) MIL-53(Al)-*0.25*NH_2*0.75*CH_3, (E) MIL-53(Al)-*0.10*NH_2*0.90*CH_3, (F) MIL-53(Al)-*0.05*NH_2*0.95*CH_3, (G) MIL-53(Al)-CH_3 and (H) MIL-53(Al).

Linker ratio calculated from ATR-IR

The areas of the peaks were deconvoluted using the Peak analyzer tool in OriginPro 2021, with a manually-defined baseline and a Gaussian function. The ratio of $HBDC{-}NH_2$ and $HBDC{-}CH_3$ were calculated using the following equation 1 on the basis of Lamber-Beer law:

$$x_a = \frac{M_a}{M_{ref}} = \frac{\frac{M_a}{M_{ref}}}{x_a^{100\%}} = \frac{\frac{M_a}{M_{ref}}}{\frac{M_a^{100\%}}{M_{ref}^{100\%}}} = \frac{\frac{A_a}{\varepsilon_a l} \cdot \frac{\varepsilon_{ref} l}{A_{ref}}}{\frac{A_a^{100\%}}{\varepsilon_a l} \cdot \frac{\varepsilon_{ref} l}{A_{ref}^{100\%}}} = \frac{\frac{A_a}{A_{ref}}}{\frac{A_a^{100\%}}{A_{ref}^{100\%}}} \tag{1}$$

Where

M: molar concentration

A: the area calculated from Gaussian

$\boldsymbol{\varepsilon}$: the absorptivity

l: the optical path length

a: functional groups (either $-NH_2$ or $-CH_3$)

100%: the two pure linker MOFs (either MIL-53-NH_2 or MIL-53-CH_3)

ref: ref peak

Taking calculating the percentage of $H_2BDC{-}NH_2$ in the mixed linker MOFs as an example: The percentage of $H_2BDC{-}NH_2$ linker (x_{-NH_2}) in mixed linker MIL-53-$x$$NH_2$*(1-x)*$CH_3$ equals to the ratio between the molar concentration of $-NH_2$ in the mixed linker MOFs and that of the total molar of linkers.

In MIL-53-NH_2, the molars of $-NH_2$ is the same as the total molars of the linker, that being said, $x_{-NH_2}^{100\%}$ is equal to 1. Then we can divide the equation by $x_{-NH_2}^{100\%}$. Simplifying this equation furthermore, the essential components left for calculating the $H_2BDC{-}NH_2$ ratio are the areas of $-NH_2$ (A_{-NH_2}) and total linkers (A_{ref}) in the mixed linker MOFs, and the areas of $-NH_2$ ($A_{-NH_2}^{100\%}$) and total linkers ($A_{ref}^{100\%}$) in MIL-53$-NH_2$. Similarly, the same principle applies to calculationg $H_2BDC{-}CH_3$ ratio.That means IR bands serving as a reference peak which can represent total amount linkers , bands corresponding to $-NH_2$, and a band represent $-CH_3$ will allow us to continue the calculation. When selecting these

peaks, two considerations were taken into account: (i) they are isolated and neat; (ii) the sum of calculated $-NH_2$ and $-CH_3$ percentage should be close to 100%. Based on the above considerations, $\mu(OH)$ at 980-986 cm^{-1} was selected as reference peak,[1] C-N at 1255 cm^{-1} was selected to represent $-NH_2$ groups, and a band at 1209 cm^{-1} was selected to represent $-CH_3$ groups.

Table S2. Calculated $-NH_2$ and $-CH_3$ ratio from ATR-IR.

	**Area C-N**	**Area C-C**	**Area μ(OH)**	**Calculated $-NH_2$**	**Calculated $-CH_3$**	**Sum**
**MIL-53(Al)-NH_2**	8.7968	0	9.95008	100%	0%	100%
**MIL-53(Al)-*0.75*NH_2*0.25*CH_3**	11.17187	0.27207	14.03942	90%	22%	112%
**MIL-53(Al)-*0.50*NH_2*0.50*CH_3**	5.10343	0.1731	7.62823	76%	26%	102%
**MIL-53(Al)-*0.25*NH_2*0.75*CH_3**	2.12129	0.33422	6.46077	37%	61%	98%
**MIL-53(Al)-*0.10*NH_2*0.90*CH_3**	0.66724	0.2897	4.5802	16%	75%	91%
**MIL-53(Al)-*0.05*NH_2*0.95*CH_3**	0.72465	0.47848	6.7838	12%	83%	95%
**MIL-53(Al)-CH_3**	0	0.64766	7.63741	0%	100%	100%

Linker ratio calculated from elemental analysis

Table S3. Weight percentage of elements and calculated $-NH_2$ ratio from elemental analysis.

	**%C**	**%H**	**%N**	**%Al**	**N:Al**	**Calculated $-NH_2$**
**MIL-53(Al)-NH_2**	46.78	3.19	6.69	10.56	0.6335	100%
**MIL-53(Al)-*0.75*NH_2*0.25*CH_3**	46.64	3.27	5.95	10.67	0.5576	88%
**MIL-53(Al)-*0.50*NH_2*0.50*CH_3**	45.59	3.29	4.33	10.83	0.3998	63%
**MIL-53(Al)-*0.25*NH_2*0.75*CH_3**	45.43	3.34	3.43	10.96	0.3130	49%
**MIL-53(Al)-*0.10*NH_2*0.90*CH_3**	44.57	3.36	1.44	12.92	0.1115	18%
**MIL-53(Al)-*0.05*NH_2*0.95*CH_3**	44.02	3.38	1.02	13.28	0.0783	12%
**MIL-53(Al)-CH_3**	42.12	3.40	0.50	14.83	0.0337	0%

There was trace of nitrogen element detected in MIL-53(Al)-CH_3 sample, yet combined other data we have (*i.e.*, TGA and ATR-IR), we are confident that this is not due to the $H_2BDC-NH_2$ linker.

Linker ratio calculated from XPS

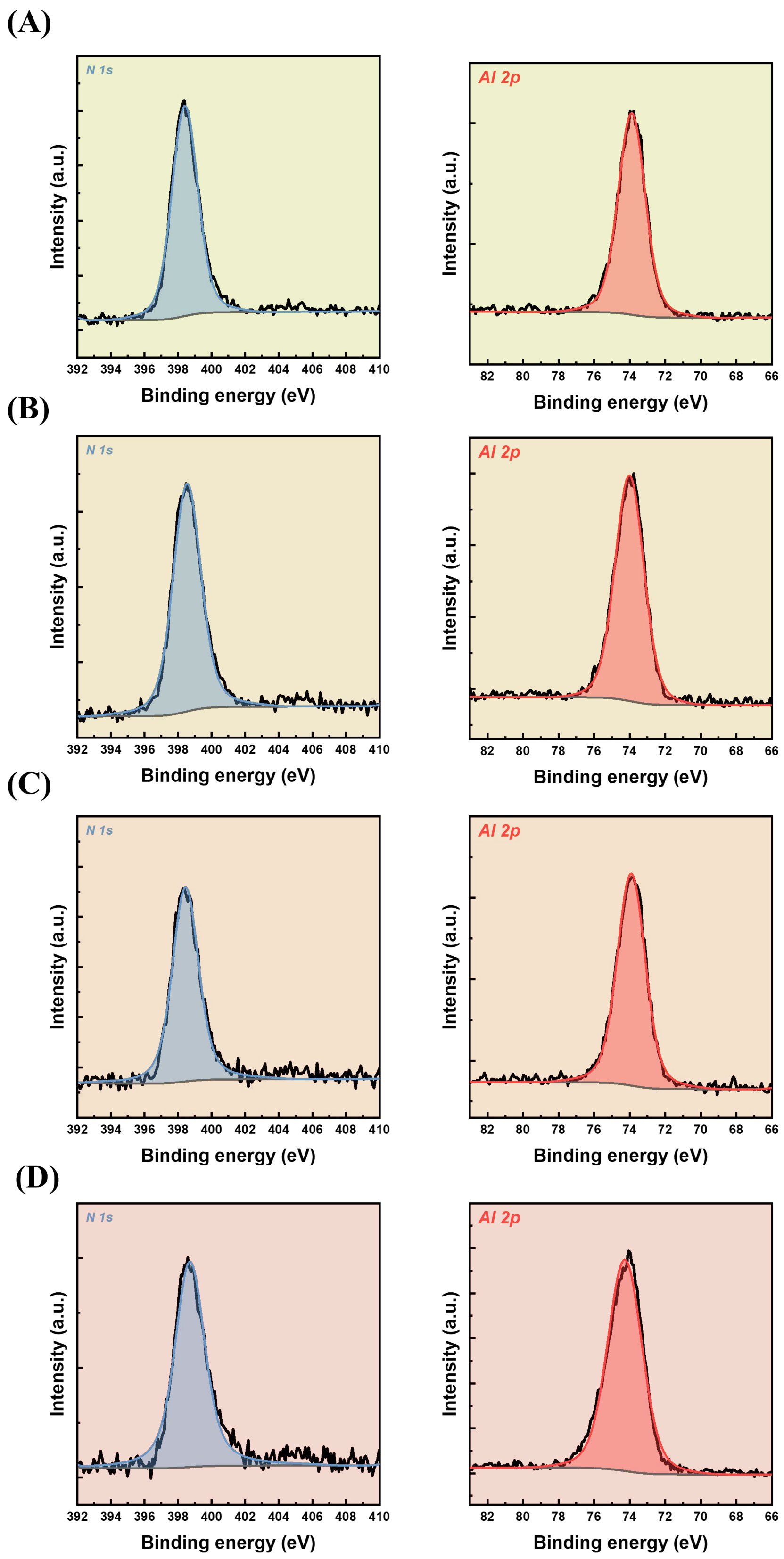

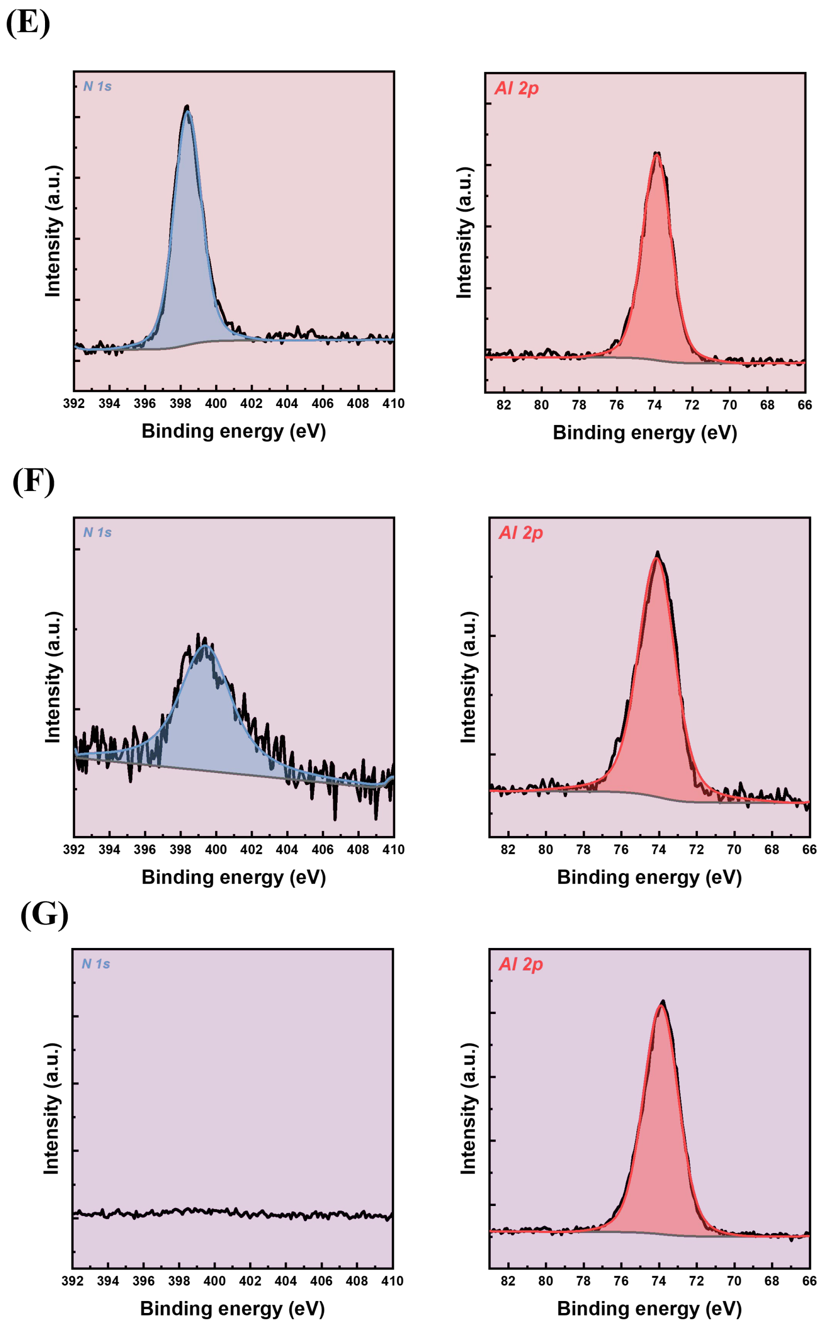

Figure S5. XPS patterns of N1s and Al2p of (A) MIL-53(Al)-NH_2,(B) MIL-53(Al)-*0.75*NH_2*0.25*CH_3,(C) MIL-53(Al)-*0.50*NH_2*0.50*CH_3,(D) MIL-53(Al)-*0.25*NH_2*0.75*CH_3,(E) MIL-53(Al)-*0.10*NH_2*0.90*CH_3,(F) MIL-53(Al)-*0.05*NH_2*0.95*CH_3, and (G) MIL-53(Al)-CH_3.

Table S4. Atomic ratios of the MIL-53(Al)-xNH$_2$*(1-x)*CH$_3$ samples from XPS analysis and calculated $-NH_2$ ratio.

	**Al at%**	**N at%**	**N:Al**	**Normalized $-NH_2$ percentage**
**MIL-53(Al)-NH$_2$**	3.73	3.51	0.941019	100%
**MIL-53(Al)-*0.75*NH$_2$*0.25*CH$_3$**	5.34	4.48	0.838951	89%
**MIL-53(Al)-*0.50*NH$_2$*0.50*CH$_3$**	3.82	2.59	0.67801	72%
**MIL-53(Al)-*0.25*NH$_2$*0.75*CH$_3$**	3.44	1.4	0.406977	43%
**MIL-53(Al)-*0.10*NH$_2$*0.90*CH$_3$**	4.41	0.57	0.129252	14%
**MIL-53(Al)-*0.05*NH$_2$*0.95*CH$_3$**	6.86	0.64	0.093294	10%
**MIL-53(Al)-CH$_3$**	6.95	0	0	0%

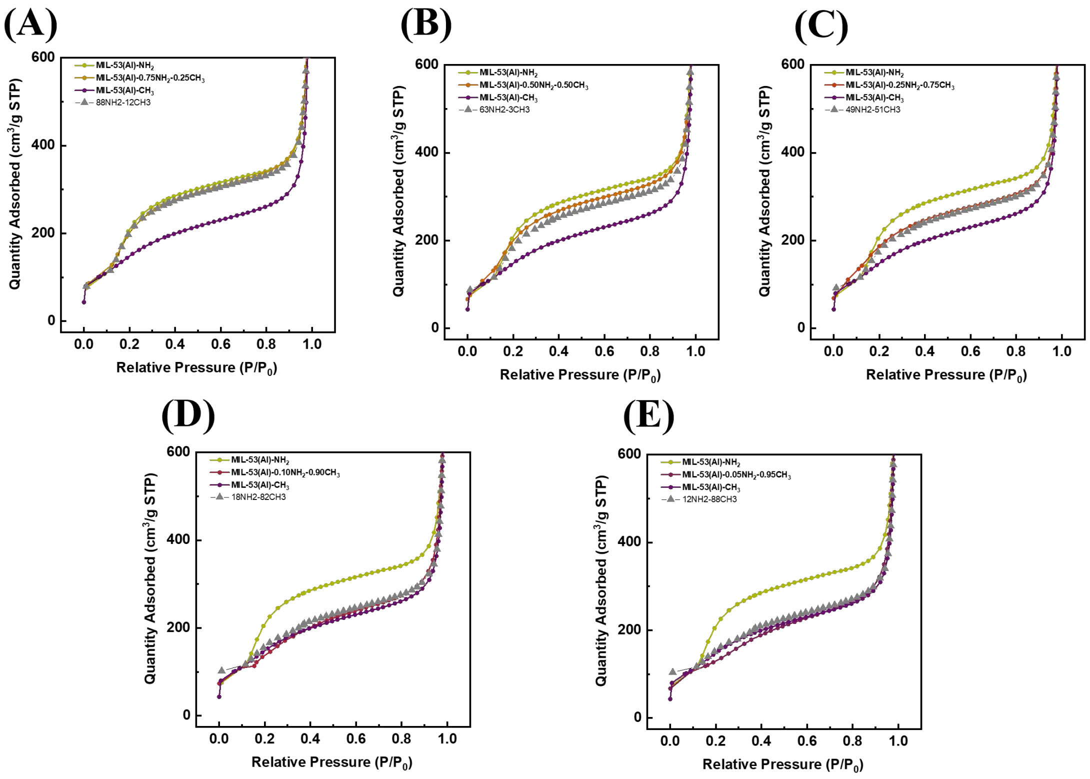

Figure S6. Weighted-average N_2 adsorption isotherms calculated based on the actual linker ratios (triangle points), compared with the experimentally measured isotherms at 77 K.

Table S5. Observed pore opening step and N_2 uptake at 0.4 P/P_0.

	Transition Step (P/P_0)	N_2 uptake (cm^3/g) at 0.4 (P/P_0)
**MIL-53(Al)-NH$_2$**	0.11	284
**MIL-53(Al)-*0.75*NH$_2$*0.25*CH$_3$**	0.12	275
**MIL-53(Al)-*0.50*NH$_2$*0.50*CH$_3$**	0.12	267
**MIL-53(Al)-*0.25*NH$_2$*0.75*CH$_3$**	0.12	250
**MIL-53(Al)-*0.10*NH$_2$*0.90*CH$_3$**	0.15	199
**MIL-53(Al)-*0.05*NH$_2$*0.90*CH$_3$**	0.15	188
**MIL-53(Al)-CH$_3$**	0.12	198
**MIL-53(Al)**	-	356

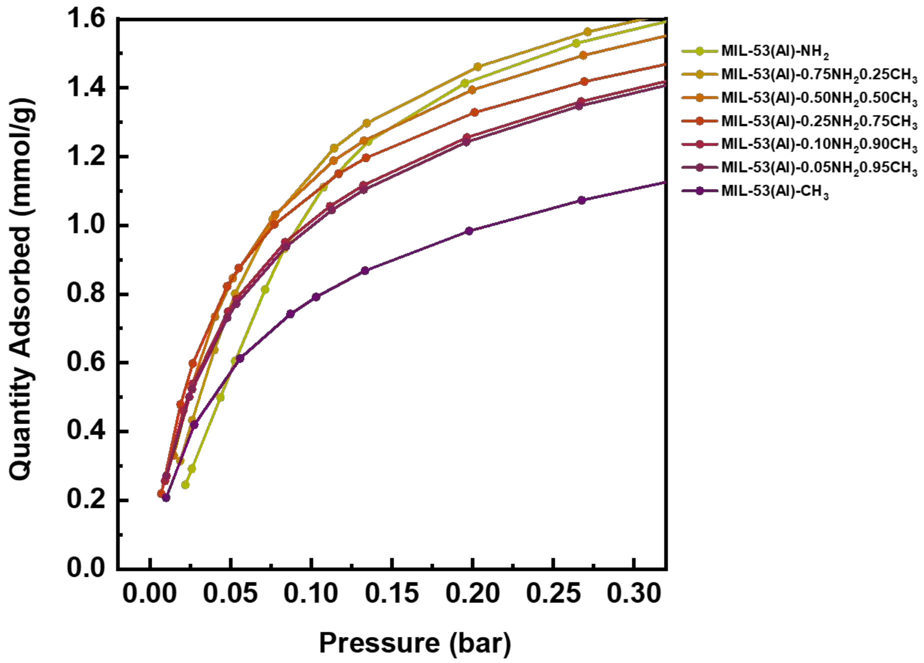

Figure S7. CO_2 adsorption isotherms at low pressure range (0-0.3 bar) at 298 K.

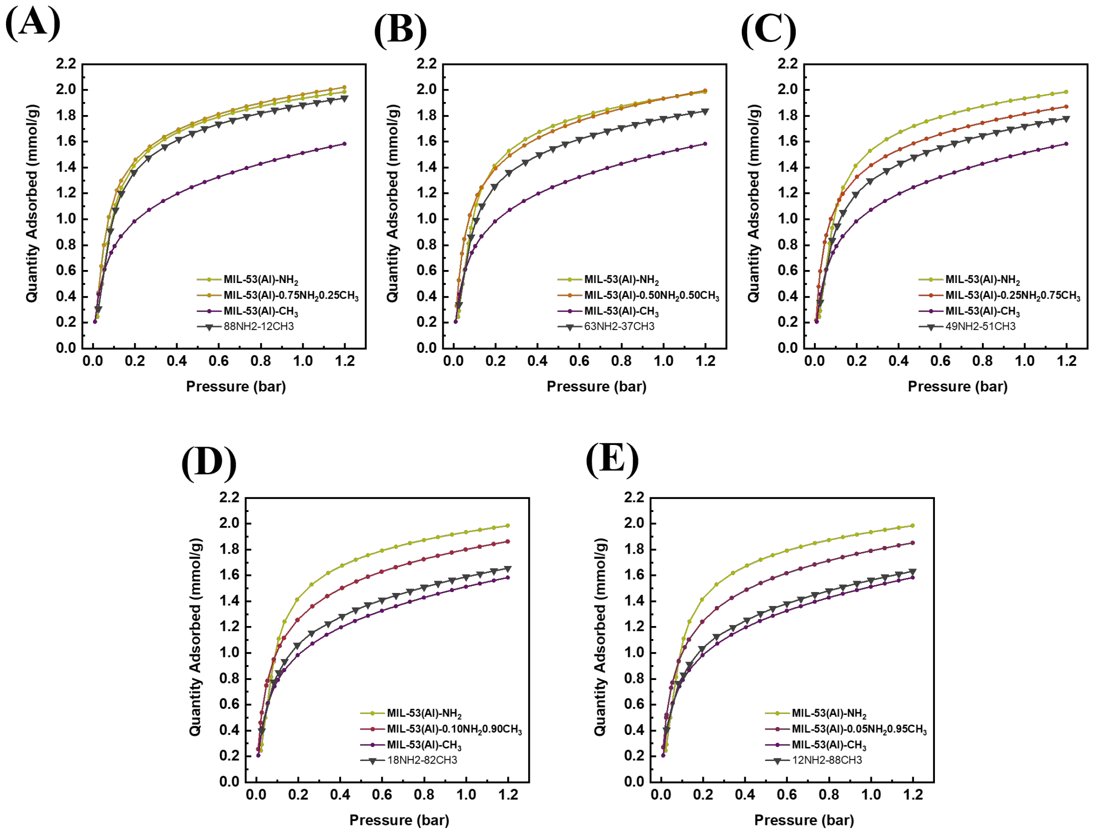

Figure S8. Weighted-average CO_2 adsorption isotherms calculated based on the actual linker ratios (triangle points), compared with the experimentally measured isotherms at 298 K.

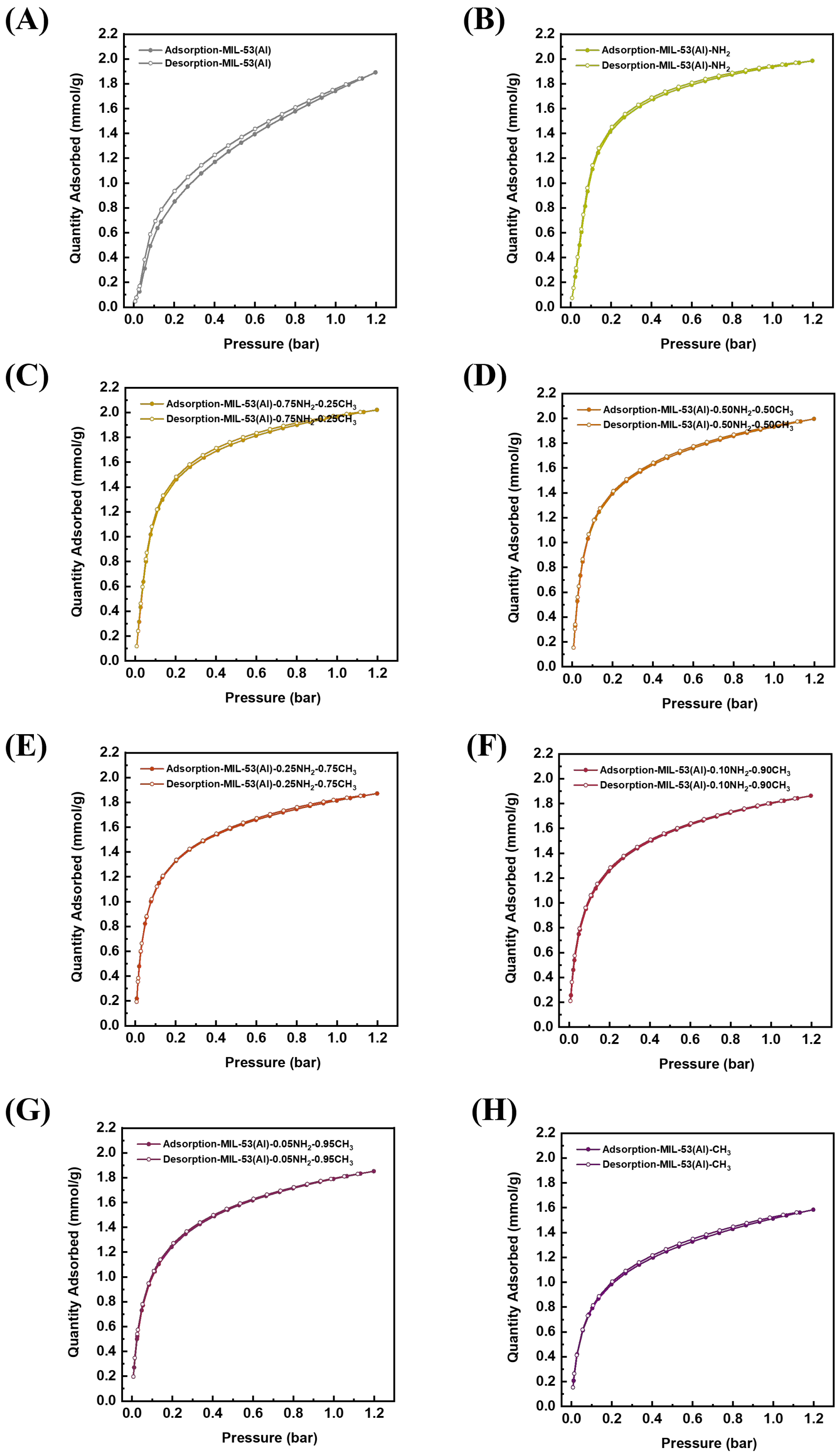

Figure S9. Individual CO_2 adsorption(filled)-desorption(empty) isotherms of (A) MIL-53(Al), (B) MIL-53(Al)-NH_2,(C) MIL-53(Al)-*0.75*NH_2*0.25*CH_3,(D) MIL-53(Al)-*0.50*NH_2*0.50*CH_3,(E) MIL-53(Al)-*0.25*NH_2*0.75*CH_3,(F) MIL-53(Al)-*0.10*NH_2*0.90*CH_3,(G) MIL-53(Al)-*0.05*NH_2*0.95*CH_3, and (H) MIL-53(Al)-CH_3 at 298 K.

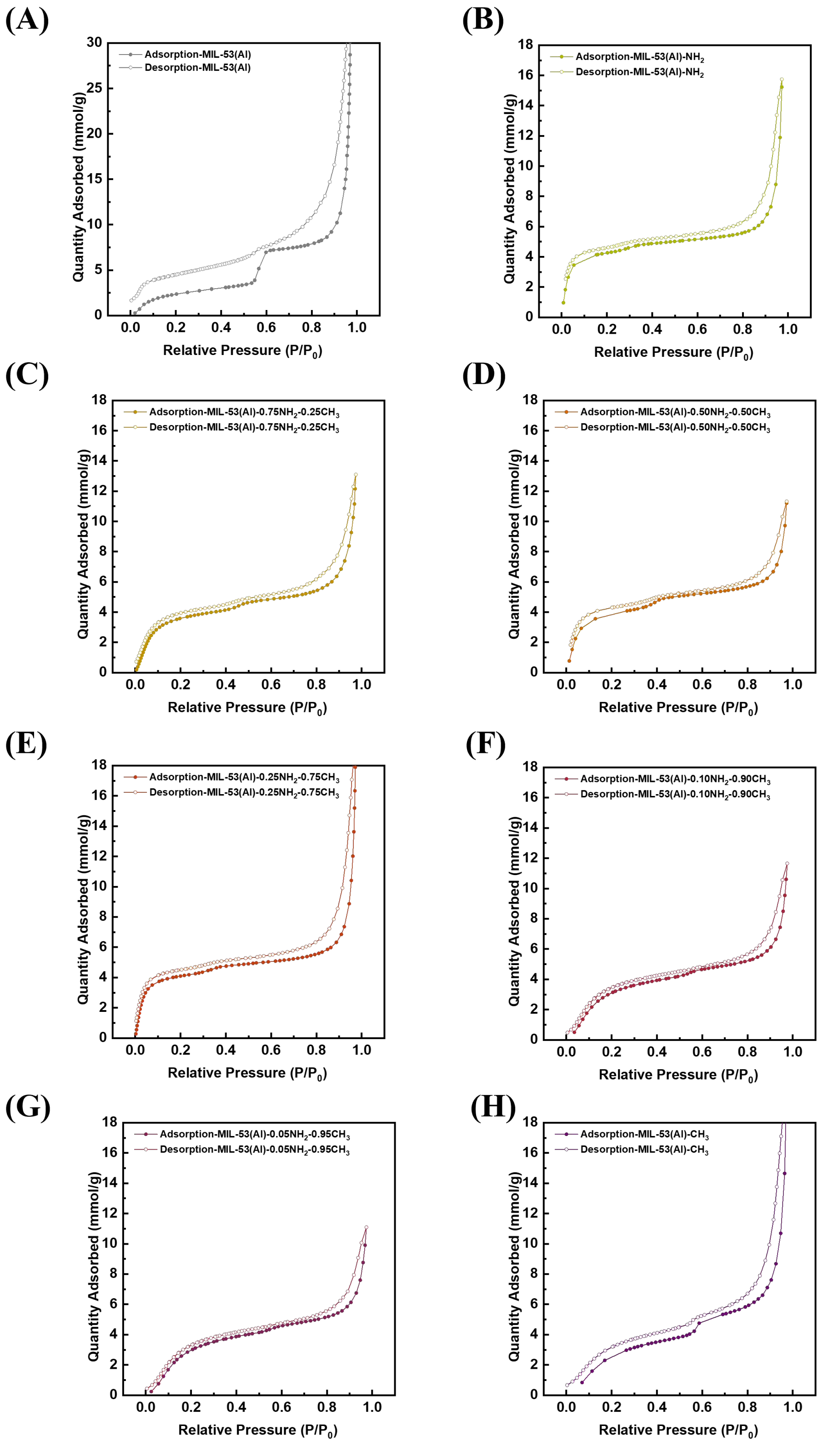

Figure S10. Individual H_2O adsorption(filled)-desorption(empty) isotherms of (A) MIL-53(Al), (B) MIL-53(Al)-NH_2,(C) MIL-53(Al)-*0.75*NH_2*0.25*CH_3,(D) MIL-53(Al)-*0.50*NH_2*0.50*CH_3,(E) MIL-53(Al)-*0.25*NH_2*0.75*CH_3,(F) MIL-53(Al)-*0.10*NH_2*0.90*CH_3,(G) MIL-53(Al)-*0.05*NH_2*0.95*CH_3, and (H) MIL-53(Al)-CH_3 at 293 K.

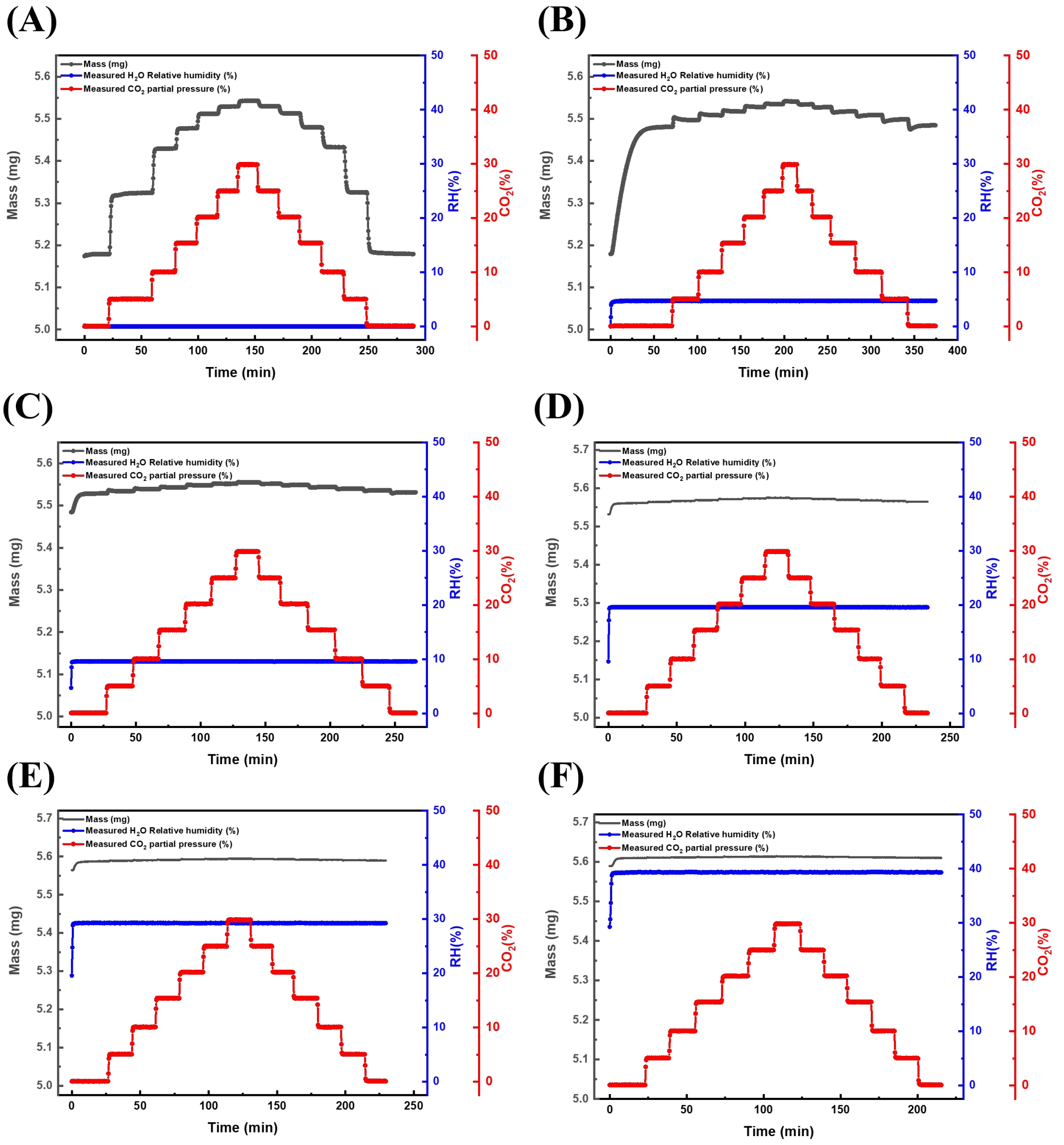

Figure S11. Kinetics of CO_2 uptake when equilibrated with (A) 0% RH, (B) 5% RH, (C) 10% RH, (D) 20% RH, (E) 30% and (F) 40% on MIL-53(Al)-NH_2 on DVS Carbon Advanced.

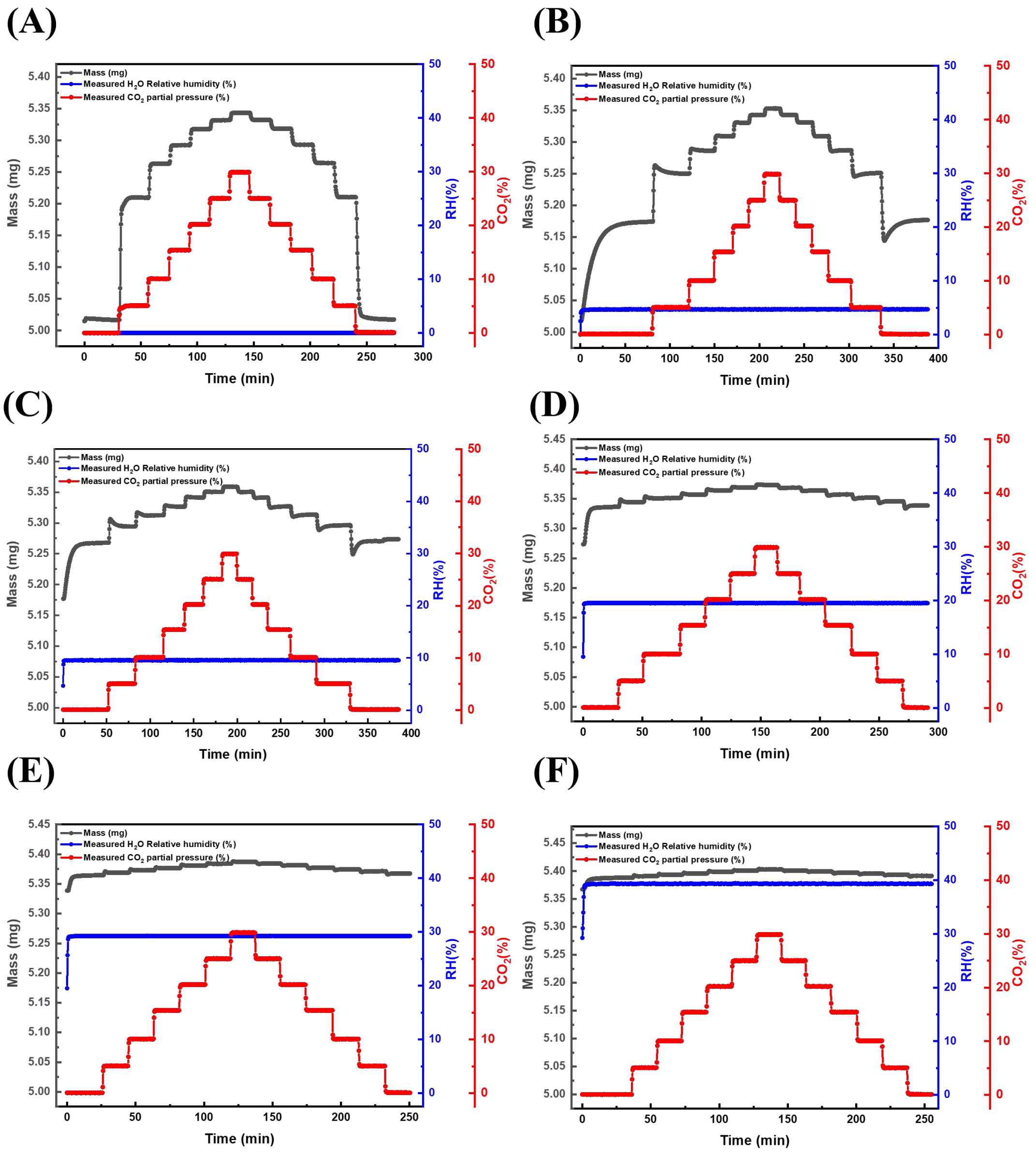

Figure S12. Kinetics of CO_2 uptake when equilibrated with (A) 0% RH, (B) 5% RH, (C) 10% RH, (D) 20% RH, (E) 30% and (F) 40% on MIL-53(Al)-*0.25*NH_2*0.75*CH_3 on DVS Carbon Advanced.

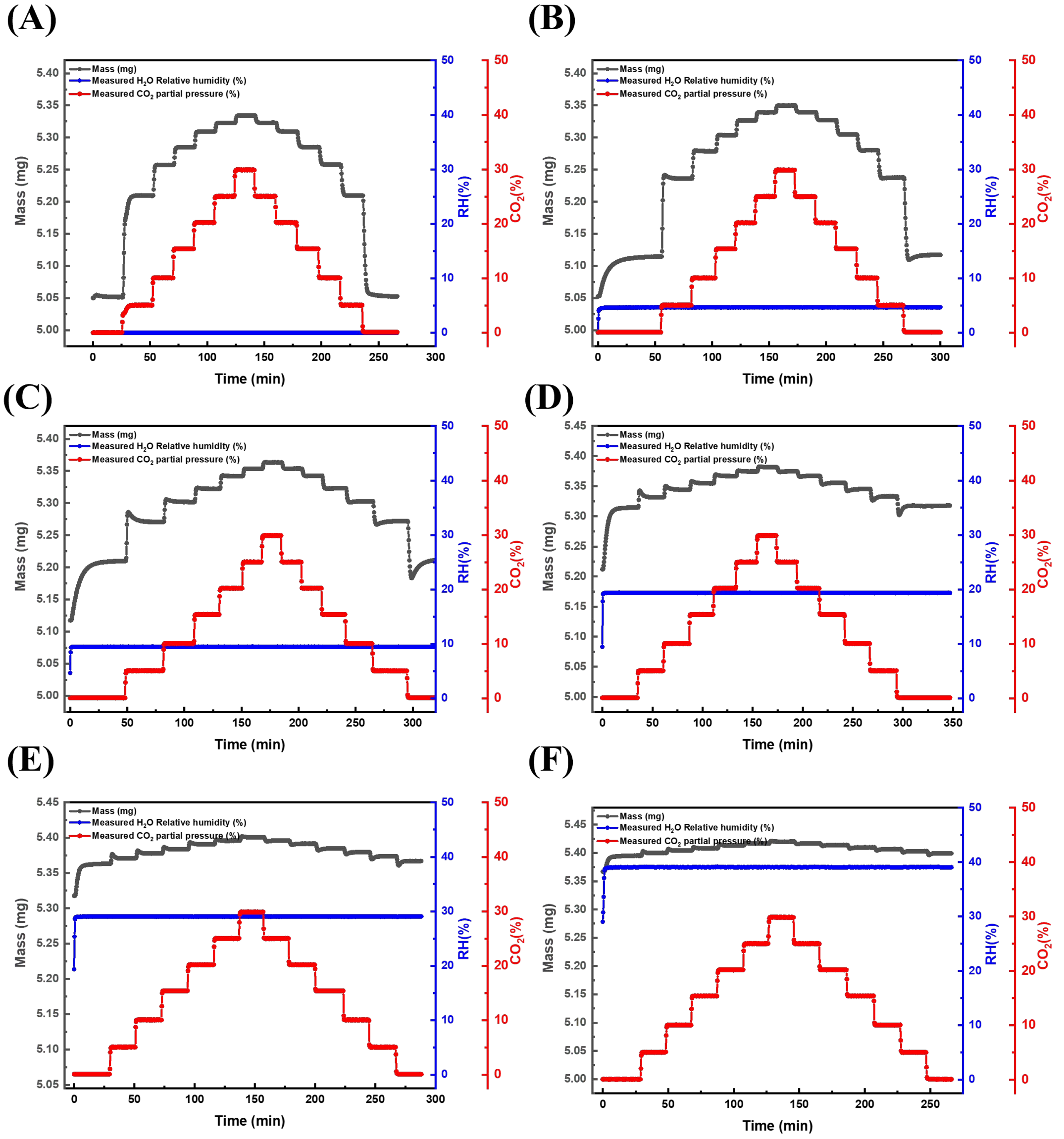

Figure S13. Kinetics of CO_2 uptake when equilibrated with (A) 0% RH, (B) 5% RH, (C) 10% RH, (D) 20% RH, (E) 30% and (F) 40% on MIL-53(Al)-*0.05*NH_2*0.95*CH_3 on DVS Carbon Advanced.

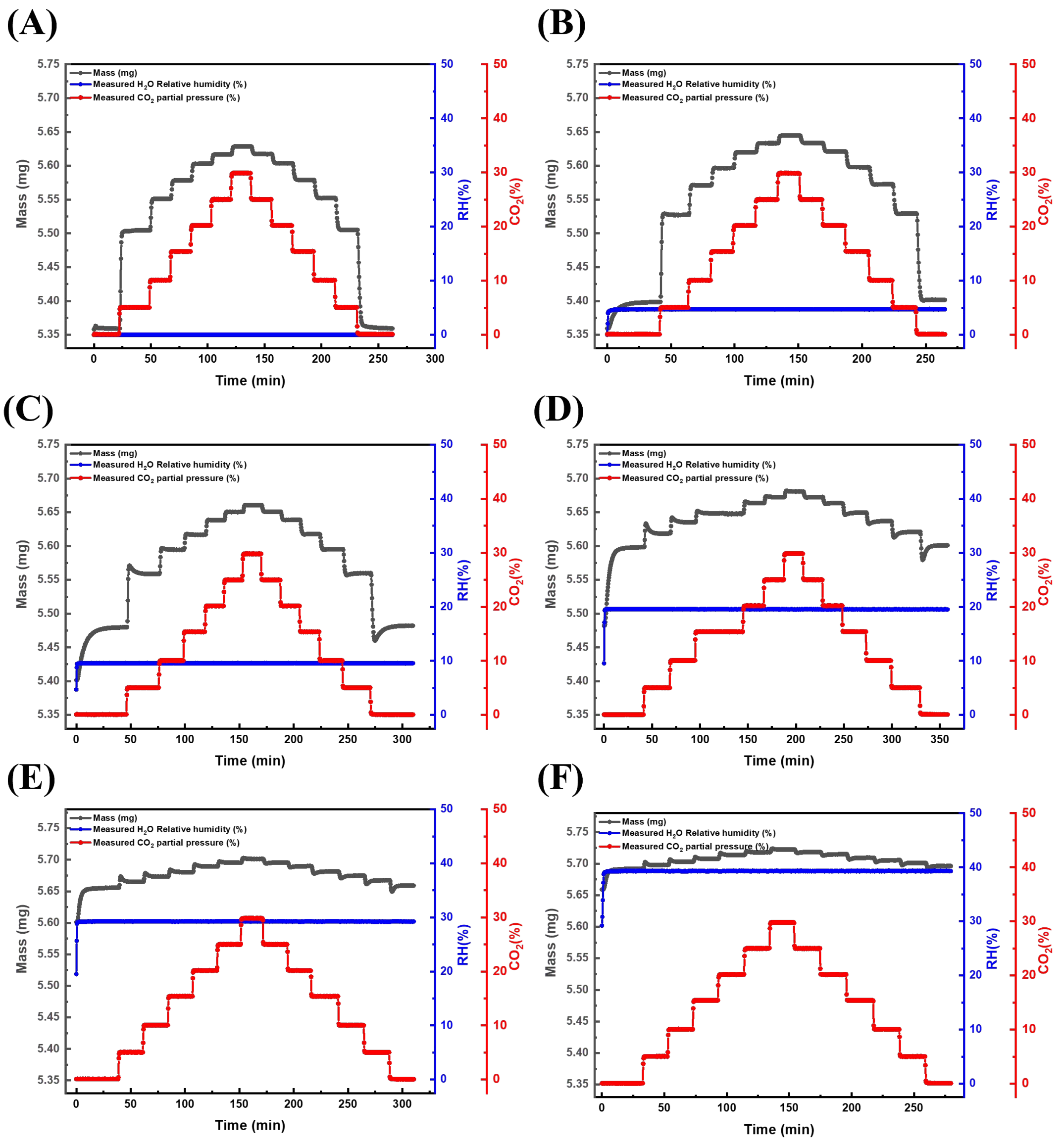

Figure S14. Kinetics of CO_2 uptake when equilibrated with (A) 0% RH, (B) 5% RH, (C) 10% RH, (D) 20% RH, (E) 30% and (F) 40% on MIL-53(Al)-CH_3 on DVS Carbon Advanced.

References

(1) Hoffman, A. E. J.; Vanduyfhuys, L.; Nevjestić, I.; Wieme, J.; Rogge, S. M. J.; Depauw, H.; Van Der Voort, P.; Vrielinck, H.; Van Speybroeck, V. Elucidating the Vibrational Fingerprint of the Flexible Metal–Organic Framework MIL-53(Al) Using a Combined Experimental/Computational Approach. *The Journal of Physical Chemistry C* **2018**, *122*, 2734–2746.
